# Supplementary material for: Large-scale cluster quantum microcombs
Source: Light Sci Appl. 2025 Apr 16;14:164. doi: 10.1038/s41377-025-01812-2 (PMC12003720; doi:10.1038/s41377-025-01812-2)
Supplement: Supplementary file 1 — Supplimentary Information for Large-scale cluster quantum microcombs [file 41377_2025_1812_MOESM1_ESM.pdf]

Supplementary Information: Large-scale cluster quantum microcombs

Ze Wang,[1,*] Kangkang Li,[1,*,†] Yue Wang,[1,*] Xin Zhou,[2] Yinke Cheng,[1,2] Boxuan Jing,[1] Fengxiao Sun,[1] Jincheng Li,[2] Zhilin Li,[2] Bingyan Wu,[1] Qihuang Gong,[1,3,4,5] Qiongyi He,[1,3,4,5†] Bei-Bei Li,[2,†] and Qi-Fan Yang[1,3,4,5†]

[1]State Key Laboratory for Artificial Microstructure and Mesoscopic Physics and Frontiers Science Center for Nano-optoelectronics School of Physics, Peking University, Beijing, 100871, China

[2]Beijing National Laboratory for Condensed Matter Physics, Institute of Physics, Chinese Academy of Sciences, Beijing, 100190, China

[3]Collaborative Innovation Center of Extreme Optics, Shanxi University, Taiyuan, 030006, China

[4]Peking University Yangtze Delta Institute of Optoelectronics, Nantong, Jiangsu, 226010, China

[5]Hefei National Laboratory, Hefei, 230088, China

[*]These authors contributed equally to this work.

[†]Corresponding author: kangkangli@pku.edu.cn; qiongyihe@pku.edu.cn; libeibei@iphy.ac.cn; leonardoyoung@pku.edu.cn.

CONTENTS

I. Theoretical Model 3
A. Equations of motion 3
B. Calculation of covariance matrix 4
C. Verification of inseparability 6
D. Computation of cluster states 7
1. Graph structures and nullifiers 7
2. Nullifiers with finite squeezing 7
3. Realignment of qumode phases 8
4. Impact of parasitic nonlinear optical processes 11
5. Determining graph structure 14
E. Optimal pumping strategy 17

II. Experimental Details 18
A. Device fabrication 18
B. Characterization of dispersion and linewidth 18
C. Experimental setup 18
D. Phase-locked balanced homodyne detection 20
E. Covariance matrix measurement 20

III. Additional Measurements 21
A. Raw squeezing of EPR pairs 21
B. Zero check of covariance matrix 21
C. Realignment of qumode phases: experiment 22

IV. Quantum Fisher Information 24

References 25

I. THEORETICAL MODEL

A. Equations of motion

We consider a longitudinal mode family of the microresonator, with resonant frequencies for modes indexed by k given by $\omega_o + D_1 k + \frac{D_2}{2}k^2 + \mathcal{O}(k^3)$ where D_1 is the free-spectral range, D_2 represents the second-order dispersion of the mode family, and ω_o is the frequency of the central mode. We define qumodes on an equidistant frequency lattice as $\omega_k = \omega_o + kD_1$. In our analysis, the mode index k ranges from k_0 to $k_0 + N - 1$, where N is the total number of qumodes considered. Note that the frequency of the qumodes and resonant modes are different due to dispersion. The field operator for each qumode is expressed as $A_k + \hat{a}_k$, where the classical field is represented by the complex amplitude A_k and the quantum fluctuation is described by the annihilation operator $\hat{a}_k$. For non-pump qumodes operated below the lasing threshold, the classical field amplitude satisfies $A_k = 0$.

Using the coupled mode equations for Kerr microresonators [1] and applying linearization, the equation of motion for $\hat{a}_k$ is derived as

$$\begin{aligned}\frac{d\hat{a}_k}{dt} &= -\left(\frac{\kappa}{2} + i\zeta_k\right)\hat{a}_k \\ &+ ig\sum_{mnp}\delta(m-n+p-k)A_m A_p \hat{a}_n^\dagger \\ &+ ig\sum_{mnp}\delta(m-n+p-k)\left(A_n^* A_p \hat{a}_m + A_n^* A_m \hat{a}_p\right) \\ &+ \sqrt{\kappa_0}\hat{V}_{0,k} + \sqrt{\kappa_e}\hat{V}_{e,k}\end{aligned} \tag{S1}$$

where g is the nonlinear coupling coefficient, and κ, κ_0, and κ_e denote the total, intrinsic, and external coupling loss rates of the microresonator, respectively, with a relation $\kappa = \kappa_0 + \kappa_e$. The function $\delta(m-n+p-k)$ is the Kronecker delta, equal to 1 if $m-n+p = k$ and 0 otherwise. The frequency detuning between the k-th qumode and the nearest microresonator mode is given by $\zeta_k = \zeta_0 + \frac{1}{2}D_2 k^2$, which accounts for the pump-cavity detuning at the zeroth mode (ζ_0) and the second-order dispersion of the mode family (D_2). The second term on the right-hand side of Eq. S1 describes two-mode squeezing (TMS), while the third term accounts for Bragg scattering (BS) and cross-phase modulation.

The vacuum fluctuations associated with the intrinsic and external coupling loss channels are represented by the Langevin operators $\hat{V}_{0,k}$ and $\hat{V}_{e,k}$, respectively. These operators are fully uncorrelated and satisfy the normalization conditions:

$$[\hat{V}_{0,k}(t), \hat{V}_{0,k'}^\dagger(t')] = [\hat{V}_{e,k}(t), \hat{V}_{e,k'}^\dagger(t')] = \delta_{k,k'}\delta(t-t'), \tag{S2}$$

$$[\hat{V}_{0,k}^\dagger(t), \hat{V}_{0,k'}(t')] = [\hat{V}_{e,k}^\dagger(t), \hat{V}_{e,k'}(t')] = 0, \tag{S3}$$

$$[\hat{V}_{0,k}(t), \hat{V}_{0,k'}(t')] = [\hat{V}_{e,k}(t), \hat{V}_{e,k'}(t')] = 0, \tag{S4}$$

$$[\hat{V}_{0,k}(t), \hat{V}_{e,k'}^\dagger(t')] = [\hat{V}_{0,k}^\dagger(t), \hat{V}_{e,k'}(t')] = [\hat{V}_{0,k}(t), \hat{V}_{e,k'}(t')] = 0. \tag{S5}$$

The set of equations in Eq. S1 can be rewritten in matrix form:

$$\frac{d}{dt}\begin{pmatrix}\hat{a}_{k_o}\\ \vdots \\ \hat{a}_k \\ \vdots \\ \hat{a}_{k_o+N-1} \\ \hat{a}_{k_o}^\dagger \\ \vdots \\ \hat{a}_k^\dagger \\ \vdots \\ \hat{a}_{k_o+N-1}^\dagger\end{pmatrix} = J\begin{pmatrix}\hat{a}_{k_o}\\ \vdots \\ \hat{a}_k \\ \vdots \\ \hat{a}_{k_o+N-1} \\ \hat{a}_{k_o}^\dagger \\ \vdots \\ \hat{a}_k^\dagger \\ \vdots \\ \hat{a}_{k_o+N-1}^\dagger\end{pmatrix} + \sqrt{\kappa_0}\begin{pmatrix}\hat{V}_{0,k_o}\\ \vdots \\ \hat{V}_{0,k} \\ \vdots \\ \hat{V}_{0,k_o+N-1} \\ \hat{V}_{0,k_o}^\dagger \\ \vdots \\ \hat{V}_{0,k}^\dagger \\ \vdots \\ \hat{V}_{0,k_o+N-1}^\dagger\end{pmatrix} + \sqrt{\kappa_e}\begin{pmatrix}\hat{V}_{e,k_o}\\ \vdots \\ \hat{V}_{e,k} \\ \vdots \\ \hat{V}_{e,k_o+N-1} \\ \hat{V}_{e,k_o}^\dagger \\ \vdots \\ \hat{V}_{e,k}^\dagger \\ \vdots \\ \hat{V}_{e,k_o+N-1}^\dagger\end{pmatrix} \tag{S6}$$

where the matrix J is composed of interaction coefficients as defined in Eq. S1:

$$J = \begin{pmatrix} R & P \\ P^* & R^* \end{pmatrix}, \quad R_{k'n'} = -\delta_{k,n}\left(\frac{\kappa}{2} + i\zeta_k\right) + ig\sum_{mp}\delta(m-p+n-k)(A_m^* A_p + A_p^* A_m),$$
$$P_{k'n'} = ig\sum_{mp}\delta(m-n+p-k)A_m A_p. \tag{S7}$$

Here matrix indices are related to mode indices by $k' = k - k_o + 1$ and $n' = n - k_o + 1$.

Two cases are considered in this work, with their corresponding equations of motion presented as examples:

- **Pump lines applied to qumodes 0 and -1:** The equations of motion are expressed as:

$$\begin{aligned} \frac{d\hat{a}_k}{dt} = &-\left(\frac{\kappa}{2} + i\zeta_k - 2ig|A_0|^2 - 2ig|A_{-1}|^2\right)\hat{a}_k \\ &+ ig\left(A_0^2\hat{a}^\dagger_{-k} + 2A_0A_{-1}\hat{a}^\dagger_{-k-1} + A_{-1}^2\hat{a}^\dagger_{-k-2}\right) \\ &+ 2ig\left(A_0^*A_{-1}\hat{a}_{k+1} + A_{-1}^*A_0\hat{a}_{k-1}\right) \\ &+ \sqrt{\kappa_0}\hat{V}_{0,k} + \sqrt{\kappa_e}\hat{V}_{e,k} \end{aligned} \tag{S8}$$

  where the second term represents TMS interactions and the third term accounts for BS.

- **Pump lines applied to qumodes -1, 0, and 1:** The equations of motion are given by:

$$\begin{aligned} \frac{d\hat{a}_k}{dt} = &-\left(\frac{\kappa}{2} + i\zeta_k - 2ig\left(|A_{-1}|^2 + |A_0|^2 + |A_1|^2\right)\right)\hat{a}_k \\ &+ ig\Big[\left(A_0^2 + 2A_{-1}A_1\right)\hat{a}^\dagger_{-k} + 2A_0A_{-1}\hat{a}^\dagger_{-k-1} \\ &\quad + A_{-1}^2\hat{a}^\dagger_{-k-2} + 2A_0A_1\hat{a}^\dagger_{-k+1} + A_1^2\hat{a}^\dagger_{-k+2}\Big] \\ &+ 2ig\left[\left(A_0^*A_{-1} + A_1^*A_0\right)\hat{a}_{k+1} + \left(A_{-1}^*A_0 + A_0^*A_1\right)\hat{a}_{k-1} \right. \\ &\quad \left. + A_1^*A_{-1}\hat{a}_{k+2} + A_{-1}^*A_1\hat{a}_{k-2}\right] \\ &+ \sqrt{\kappa_0}\hat{V}_{0,k} + \sqrt{\kappa_e}\hat{V}_{e,k} \end{aligned} \tag{S9}$$

  where the second term describes TMS, and the third term accounts for BS.

The conditions that lased optical fields are at qumodes other than pump lines, are out of consideration in the above equations.

B. Calculation of covariance matrix

The covariance of two quadratures $\hat{q}_m$ and $\hat{q}'_n$ is defined as

$$\mathrm{Cov}(\hat{q}_m, \hat{q}'_n) = \frac{1}{2}(\langle\hat{q}_m\hat{q}'_n\rangle + \langle\hat{q}'_n\hat{q}_m\rangle) - \langle\hat{q}_m\rangle\langle\hat{q}'_n\rangle \tag{S10}$$

where m and n denote mode indices, and $\langle\cdot\rangle$ represent expectation values. The quadrature operators $\hat{q}$ and $\hat{q}'$ can be chosen as either $\hat{x} = (\hat{a} + \hat{a}^\dagger)/\sqrt{2}$ or $\hat{p} = -i(\hat{a} - \hat{a}^\dagger)/\sqrt{2}$. Provided $\langle\hat{q}_m\rangle = 0$, this expression is equivalent to

$$\mathrm{Cov}(\hat{q}_m, \hat{q}'_n) = \left\langle\left(\frac{1}{\sqrt{2}}\left(\hat{q}_m + \hat{q}'_n\right)\right)^2\right\rangle - \frac{1}{2}\langle\hat{q}_m^2\rangle - \frac{1}{2}\langle\hat{q}'^{\,2}_n\rangle \tag{S11}$$

which can be experimentally determined via balanced homodyne detection.

The covariance matrix σ characterized a Gaussian quantum state of a collection of qumodes is comprised of different covariances, which is organized in this text as follows

$$\sigma = \begin{pmatrix} \ddots & & & \ddots & & \\ & \mathrm{Cov}(\hat{x}_m, \hat{x}_n) & & & \mathrm{Cov}(\hat{x}_m, \hat{p}_n) & \\ & & \ddots & & & \ddots \\ \ddots & & & \ddots & & \\ & \mathrm{Cov}(\hat{p}_m, \hat{x}_n) & & & \mathrm{Cov}(\hat{p}_m, \hat{p}_n) & \\ & & \ddots & & & \ddots \end{pmatrix} = \begin{pmatrix} \sigma_{xx} & \sigma_{xp} \\ \sigma_{px} & \sigma_{pp} \end{pmatrix} \tag{S12}$$

To compute the covariance matrix of the quantum state generated in our system, it is necessary to solve Eq. S1. We express the annihilation (creation) operator $\hat{a}_k$ ($\hat{a}_k^\dagger$) in the frequency domain as

$$\hat{a}_k(t) = \int \tilde{a}_k(\omega) e^{i\omega t}\,\mathrm{d}\omega, \quad \hat{a}_k^\dagger(t) = \int \tilde{a}_k^\dagger(-\omega) e^{i\omega t}\,\mathrm{d}\omega$$

such that the Fourier transform of Eq. S1 yields

$$\overline{a}(\omega) = \sqrt{\kappa_e} M(\omega) \overline{V}_e(\omega) + \sqrt{\kappa_0} M(\omega) \overline{V}_0(\omega) \tag{S13}$$

where

$$\overline{a}(\omega) = \begin{pmatrix} \vdots \\ \tilde{a}_k(\omega) \\ \vdots \\ \tilde{a}_k^\dagger(-\omega) \\ \vdots \end{pmatrix}, \quad \overline{V}_e(\omega) = \begin{pmatrix} \vdots \\ \tilde{V}_{e,k}(\omega) \\ \vdots \\ \tilde{V}_{e,k}^\dagger(-\omega) \\ \vdots \end{pmatrix}, \quad \overline{V}_0(\omega) = \begin{pmatrix} \vdots \\ \tilde{V}_{0,k}(\omega) \\ \vdots \\ \tilde{V}_{0,k}^\dagger(-\omega) \\ \vdots \end{pmatrix} \tag{S14}$$

The transfer matrix $M(\omega)$ is defined as

$$M(\omega) = (i\omega \mathrm{I} - J)^{-1} \tag{S15}$$

Note that ω corresponds to the offset frequency used in the balanced homodyne detection in the experiment, which should be chosen much smaller than the linewidth of the microresonator. In the numerical analysis presented hereafter, we set $\omega \approx 0$, and use $\tilde{a}_k(0)$ to approximately represent $\hat{a}_k(t)$.

The detected quantum states are those emitted to the waveguide coupler, which are related to the intracavity qumodes by

$$\hat{a}_{\mathrm{out},k} = \sqrt{\kappa_e}\hat{a}_k - \hat{V}_{e,k} \tag{S16}$$

Additionally, losses occur during the detection process. Incorporating the detection efficiency of the system, denoted by η_d, the state received at the detector is expressed as

$$\hat{a}_{\mathrm{det},k} = \sqrt{\eta_d}\hat{a}_{\mathrm{out},k} + \sqrt{1-\eta_d}\hat{V}_{l,k} \tag{S17}$$

where $\hat{V}_{l,k}$ represents an additional vacuum fluctuation source that is uncorrelated with $\hat{V}_{0,k}$ and $\hat{V}_{e,k}$. Consequently, the detected field operators of qumodes can be expressed as

$$\overline{a}_{\mathrm{det}} = \sqrt{\eta_d}(\kappa_e M - \mathrm{I})\overline{V}_e + \sqrt{\eta_d \kappa_e \kappa_0} M \overline{V}_0 + \sqrt{1-\eta_d}\overline{V}_l \tag{S18}$$

The quadratures of these qumodes, $\overline{q} = (\ldots, \hat{x}_k, \ldots, \hat{p}_k, \ldots)^T$, are related to the annihilation and creation operators by

$$\overline{q} = \mathrm{T}\overline{a}, \quad \mathrm{T} = \frac{1}{\sqrt{2}}\begin{pmatrix} \mathrm{I} & \mathrm{I} \\ -i\mathrm{I} & i\mathrm{I} \end{pmatrix} \tag{S19}$$

Therefore, the quadratures $\overline{q}$ can be expressed in terms of combinations of Langevin operators, $\overline{W}_{e,0,l} = \mathrm{T}\overline{V}_{e,0,l}$, as

$$\overline{q}_{\mathrm{det}} = \sqrt{\frac{\eta_d}{\kappa^2}}(\kappa_e S - \kappa_0 \mathrm{I})\overline{W}_e + \sqrt{\frac{\eta_d \kappa_e \kappa_0}{\kappa^2}}(S + \mathrm{I})\overline{W}_0 + \sqrt{1-\eta_d}\overline{W}_l, \quad S = \mathrm{T}(\kappa M - \mathrm{I})\mathrm{T}^{-1} \tag{S20}$$

Note that S is a symplectic matrix.

Utilizing the commutation relations of the vacuum fluctuation operators as outlined in Eqs. (S2)–(S5), the following relations can be derived

$$\overline{W}_e\overline{W}_e^\top = \overline{W}_0\overline{W}_0^\top = \overline{W}_l\overline{W}_l^\top = \mathrm{I}, \quad \overline{W}_e\overline{W}_0^\top = \overline{W}_e\overline{W}_l^\top = \overline{W}_0\overline{W}_l^\top = \mathbf{0} \tag{S21}$$

and the covariance matrix at the detector is given by

$$\begin{aligned} \sigma_{\mathrm{det}} &= \langle \overline{q}_{\mathrm{det}} \overline{q}_{\mathrm{det}}^\top \rangle \\ &= \frac{\eta_d}{\kappa^2}(\kappa_e S - \kappa_0 \mathrm{I})(\kappa_e S - \kappa_0 \mathrm{I})^\top + \frac{\eta_d \kappa_e \kappa_0}{\kappa^2}(S + \mathrm{I})(S + \mathrm{I})^\top + (1-\eta_d)\mathrm{I} \\ &= (1 - \eta_d \eta_e)\mathrm{I} + \eta_d \eta_e S S^\top \end{aligned} \tag{S22}$$

where the loading factor is defined as $\eta_e = \frac{\kappa_e}{\kappa}$. The total efficiency $\eta = \eta_e \eta_d$.

We present two examples of calculated covariance matrices in our system, with pump powers adopted to generate 1D and 2D cluster states separately and consistent with the experiment conditions. P_k in the following text denotes the pump power at qumode k.

- **1D cluster state:** The pump powers at qumodes 0 and 1 in the simulations are:

$$P_0 = 0.7P_{\mathrm{th}}, \quad P_{-1} = 0.2P_0 = 0.14P_{\mathrm{th}} \tag{S23}$$

  The parametric oscillation threshold $P_{\mathrm{th}} = \kappa^3/(8g\kappa_e\hbar\omega_0)$. The resulting covariance matrix is depicted in Fig. S1a. Non-zero correlations are distributed primarily on qumode pairs $(k,k)(k,k\pm1)(k,-k)(k,-k\pm1)(k,-k-2)$.

- **2D cluster state:** The pump powers at qumodes -1,0 and 1 in the simulations are:

$$P_{-1} = 0.4P_{\mathrm{th}}, \quad P_0 = 0.2P_{-1} = 0.08P_{\mathrm{th}}, \quad P_0 = 0.16P_{-1} = 0.064P_{\mathrm{th}} \tag{S24}$$

  The resulting covariance matrix is shown in Fig. S1b. Non-zero correlations are distributed primarily on qumode pairs $(k,k)(k,k\pm1)(k\pm2)(k,-k)(k,-k\pm1)(k,-k\pm2)(k,-k-3)$.

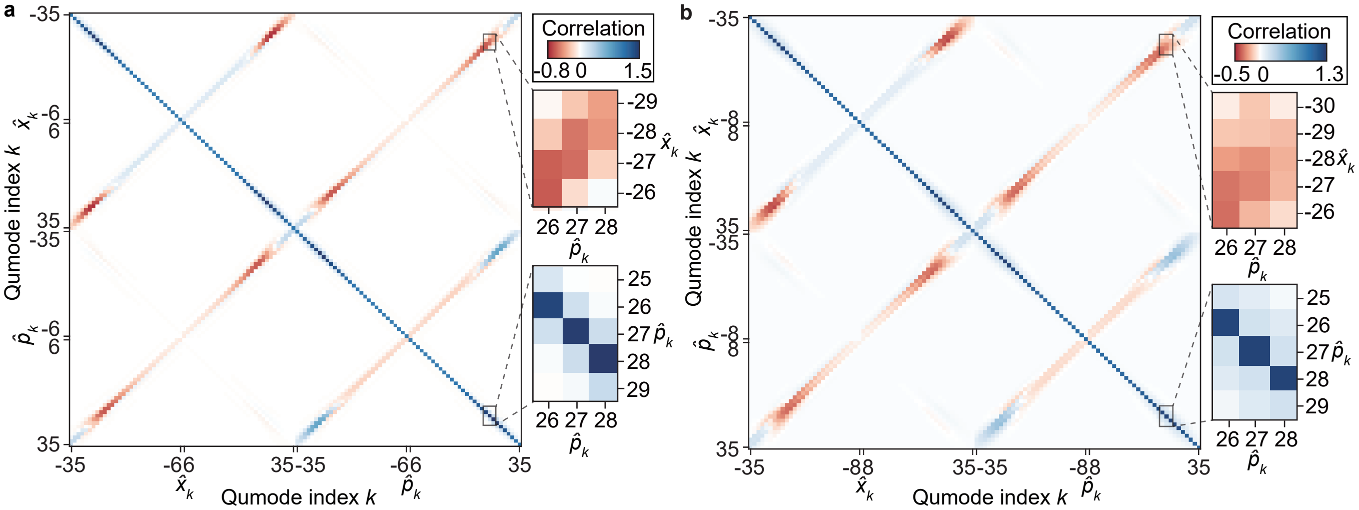

Fig. S1. **Simulated covariance matrices. a,** Simulated 60-mode covariance matrix for pump lines applied to qumodes 0 and 1. **b,** Simulated 56-mode covariance matrix for pump lines applied to qumodes -1, 0 and 1. Parameters in the simulations are: $D_2 = 0.0026\kappa$, $\zeta_0 = -0.75\kappa$, $\eta_e = \eta_d = 1$.

C. Verification of inseparability

The inseparability of a quantum state is verified by applying the positive partial transpose (PPT) criterion to its covariance matrix σ. This procedure begins by partitioning the N-mode quantum state into two subsets: part A containing n modes and part B containing $N-n$ modes. The partial transpose is performed by inverting the $\hat{p}$ quadratures of all modes in either set A or set B, thereby transforming the original covariance matrix σ into a partially transposed matrix σ_{PPT}

$$\sigma_{\mathrm{PPT}} = t_{A|B}\cdot\sigma\cdot t_{A|B}, \quad t_{A|B} = \mathrm{diag}\{\overbrace{1,...,1}^{\hat{x}}\,|\,\overbrace{\underbrace{1,..,1}_{A},\underbrace{-1,...,-1}_{B}}^{\hat{p}}\} \tag{S25}$$

The quantum state is inseparable across the bipartition A—B if at least one symplectic eigenvalue ν_k of σ_{PPT}, obtained via the symplectic decomposition $\sigma_{\mathrm{PPT}} = S^T\nu S$ with $\nu = \mathrm{diag}(\ldots,\nu_k,\ldots)$, satisfies

$$\nu_k < 1 \tag{S26}$$

where the symplectic eigenvalues are ordered such that $\nu_1 \leq \nu_2 \leq \ldots$. In other words, the minimum symplectic eigenvalue $\nu_{\min}$ (also referred to as the PPT value) being less than one indicates inseparability for the corresponding bipartition. Consequently, if all $2^{N-1}-1$ possible bipartitions of an N-mode state exhibit a PPT value below one, the state is considered completely inseparable.

D. Computation of cluster states

1. *Graph structures and nullifiers*

A cluster state offers a graph-based framework to represent multi-mode entanglement, with the edges of the graph corresponding to two-mode correlations. This structure is mathematically defined by a real, symmetric adjacency matrix $\mathbf{A}$. For a continuous-variable (CV) cluster state $|\psi_{\mathbf{A}}\rangle$, the adjacency matrix $\mathbf{A}$ satisfies the following relation:

$$(\overline{p} - \mathbf{A}\overline{x})\,|\psi_{\mathbf{A}}\rangle = \mathbf{0} \tag{S27}$$

Here, $\overline{p} = (\hat{p}_1, \ldots, \hat{p}_N)^\top$ and $\overline{x} = (\hat{x}_1, \ldots, \hat{x}_N)^\top$ denote the vectors of momentum and position quadrature operators, respectively, with N representing the number of qumodes. This equation establishes that the cluster state $|\psi_{\mathbf{A}}\rangle$ is an eigenstate of N linear combinations of quadrature operators, expressed as $\hat{p}_k - \sum_n A_{kn}\hat{x}_n$ for $k = 1, \ldots, N$, each having an eigenvalue of zero. These operators, known as *nullifiers* and denoted $\{\hat{N}_k\}$, form an orthogonal set. The nullifiers' vanishing variance reflects infinite squeezing, a defining feature of ideal cluster states.

Non-zero elements of the adjacency matrix A_{kn} represent quantum correlations between qumode pairs (k, n). In the corresponding graph representation, these correlations are visualized as edges connecting nodes k and n, with edge weights given by A_{kn}. This graph provides an intuitive depiction of the cluster state's quantum correlations and nullifiers, offering a clear framework to understand its entanglement properties.

2. *Nullifiers with finite squeezing*

In practical systems, infinite squeezing is unattainable. As a result, approximate nullifiers with finite squeezing are employed. For a given set of approximate nullifiers $\hat{r}$, the covariance matrix is defined as:

$$\mathrm{Cov}[\hat{r}] = \langle \hat{r}\hat{r}^\top \rangle = \overline{t}\langle \overline{q}\,\overline{q}^\top \rangle \overline{t}^\top = \overline{t}\,\sigma\,\overline{t}^\top, \quad \hat{r} = \overline{t}\,\overline{q} \tag{S28}$$

Where $\overline{q}$ denotes the vector of quadrature operators, and $\overline{t}$ is the transformation matrix, the noise variances of the nullifiers are given by the diagonal elements of $\mathrm{Cov}[\hat{r}]$. To assess the quality of the nullifiers, the trace of $\mathrm{Cov}[\hat{r}]$, denoted as $\mathrm{tr}\,[\mathrm{Cov}[\hat{r}]]$, is commonly used as a measure of residual error. The objective is to identify the set of nullifiers that minimizes this residual error.

In the ideal scenario of perfect efficiency ($\eta = 1$), the covariance matrix expression in Eq. (S22) simplifies to

$$\sigma = SS^\top \tag{S29}$$

Utilizing the intrinsic decomposition of the symplectic matrix S,

$$S = \begin{pmatrix} \mathrm{I} & \mathbf{0} \\ \mathbf{V} & \mathrm{I} \end{pmatrix} \begin{pmatrix} \mathbf{U}^{-1/2} & \mathbf{0} \\ \mathbf{0} & \mathbf{U}^{1/2} \end{pmatrix} \mathbf{X} \tag{S30}$$

where $\mathbf{X}\mathbf{X}^\top = \mathrm{I}$ and $\mathbf{V}, \mathbf{U}$ are symmetric, the covariance matrix σ_{p} is decomposed into

$$\sigma = \begin{pmatrix} \mathbf{U}^{-1} & \mathbf{U}^{-1}\mathbf{V} \\ \mathbf{V}\mathbf{U}^{-1} & \mathbf{U} + \mathbf{V}\mathbf{U}^{-1}\mathbf{V} \end{pmatrix} \tag{S31}$$

It is figured that $\mathrm{Cov}[(\overline{p} - \mathbf{V}\overline{x})]$ based on the matrix component $\mathbf{V}$ in σ (Eq. (S31)) induces exactly another matrix component $\mathbf{U}$ in σ:

$$\mathrm{Cov}[(\overline{p} - \mathbf{V}\overline{x})] = \mathbf{U} \tag{S32}$$

The correspondence between Eq. (S27) and Eq. (S32) facilitates a direct extraction of approximate nullifiers from σ:

$$\mathbf{V} = \sigma_{xx}^{-1} \cdot \sigma_{xp}, \quad \mathbf{U} = \sigma_{xx}^{-1} \tag{S33}$$

with $\mathbf{V}$ as approximate adjacency matrix and the trace of $\mathbf{U}$ as the residual error. The approximate nullifiers $\hat{N}_k = \hat{p}_k - \sum_n V_{kn}\hat{x}_n$ with minimum residual error $\mathrm{tr}\,[\mathbf{U}]$ can be achieved by the proper choice of bases for σ. The optimization process is discussed in detail in section I D 3.

In practical systems, the efficiency $\eta < 1$, which degrades the purity of the states and derives a detected covariance matrix $\sigma_{\mathrm{det}} = (1-\eta)\mathrm{I} + \eta\sigma$. The expression of $\mathbf{V}, \mathbf{U}$ in Eq. (S31) is rewritten in the form of σ_{det}

$$\mathbf{V} = \beta^{-1}\sigma_{\mathrm{det},xx}^{-1}\sigma_{\mathrm{det},xp}, \quad \mathbf{U} = \eta\beta^{-1}\sigma_{\mathrm{det},xx}^{-1} \tag{S34}$$

where $\beta = \mathrm{I} - (1-\eta)\sigma_{\mathrm{det},xx}^{-1}$. Setting the approximate nullifiers as $\overline{p} - \mathbf{V}\overline{x}$ gives the residual error

$$\mathrm{tr}[\eta\mathbf{U} + (1-\eta)\mathrm{I} + (1-\eta)\mathbf{V}^2] \tag{S35}$$

Since the total efficiency of the system cannot be precisely obtained, experimentally we adopt another set of approximate nullifiers using measurement results as $\overline{p} - \mathbf{V}_{\mathrm{det}}\overline{x}$, with

$$\mathbf{V}_{\mathrm{det}} = \sigma_{\mathrm{det},xx}^{-1}\sigma_{\mathrm{det},xp} \tag{S36}$$

The residual error yields

$$\mathrm{tr}[\eta\mathbf{U} + (1-\eta)\mathrm{I} + (1-\eta)(\beta\mathbf{V})^2 + \eta(\beta\mathbf{V} - \mathbf{V})\mathbf{U}^{-1}(\beta\mathbf{V} - \mathbf{V})] \tag{S37}$$

The term $(1-\eta)\mathrm{I}$ in Eq. (S35) (S37) indicates that the total efficiency limits nullifier squeezing of the non-pure state.

3. *Realignment of qumode phases*

The quantum state remains invariant under a gauge transformation, wherein the phases of the qumodes are altered (i.e., transforming $\hat{a}_k$ to $\hat{a}_k e^{i\theta_k}$), which correspond to a phase shift in the LOs in the experiment. However, such a transformation changes the covariance matrix, giving rise to a new set of approximate nullifiers (still using the standard form) with a different residual error. Therefore, finding the optimal set of the qumode phases is important to determine the most approximate nullifiers with minimum residual error. Here we present two methods for realigning qumode phases below:

- **Global Optimization.** The covariance matrix is recalculated for each set of qumode phases, and the residual error is updated using Eq. S37. The optimization process is performed to all qumode phases until the error falls below a predefined threshold. The workflow of the global optimization procedure is illustrated in Fig. S2a.
- **Line-by-line Optimization.** When the graph structure of the cluster state (the connectivity) is known, the global optimization can be streamlined into a line-by-line approach. As depicted in Fig. S2d, the phases of the qumodes are adjusted in sequence to minimize the variance of $\hat{x}_k + \hat{x}_{k-1}$ for k. After the phases of all qumodes are adjusted, an additional phase shift of $-\pi/2$ (i.e., $\hat{x}$ is changed to $\hat{p}$) is assigned to every other qumode in sequence to ensure the nullifiers conform to the standard form. This approach can also be used for rapid acquisition of the nullifiers in experiments.

We present two examples below:

- **1D cluster state.**

  The primary pump line is applied to qumode 0, and a secondary pump line is applied to qumode -1, with power ratios $P_0 : P_{-1} = 1 : 0.2$. The mode indices are denoted by the subscripts. The results after the two optimization processes are presented in Fig. S2. Considering that major TMS occurs between qumode pairs $(k, -k)$ and $(k, -k-1)$, the line-by-line optimization is performed along the sequence

  $$(\ldots, k, -k, k-1, -k+1, \ldots) \tag{S38}$$

  The resulting covariance matrices are highly similar, validating the effectiveness of the line-by-line optimization approach.

- **2D cluster state.**

  The primary pump line is applied to qumode -1, and two secondary pump lines are applied to qumodes 0 and 1, with power ratios $P_{-1} : P_0 : P_1 = 1 : 0.2 : 0.16$. The simulation results for these two optimization processes are shown in Fig. S3. In this configuration, major TMS operations are applied to qumode pairs $(k, -k)$, $(k, -k-1)$, and $(k, -k-2)$. Consequently, the line-by-line optimization is performed in the following sequence (Fig. S3d):

  $$(\ldots, k, -k-1, k-1, -k, \ldots) \tag{S39}$$

  As expected, the covariance matrices obtained using both methods are very similar.

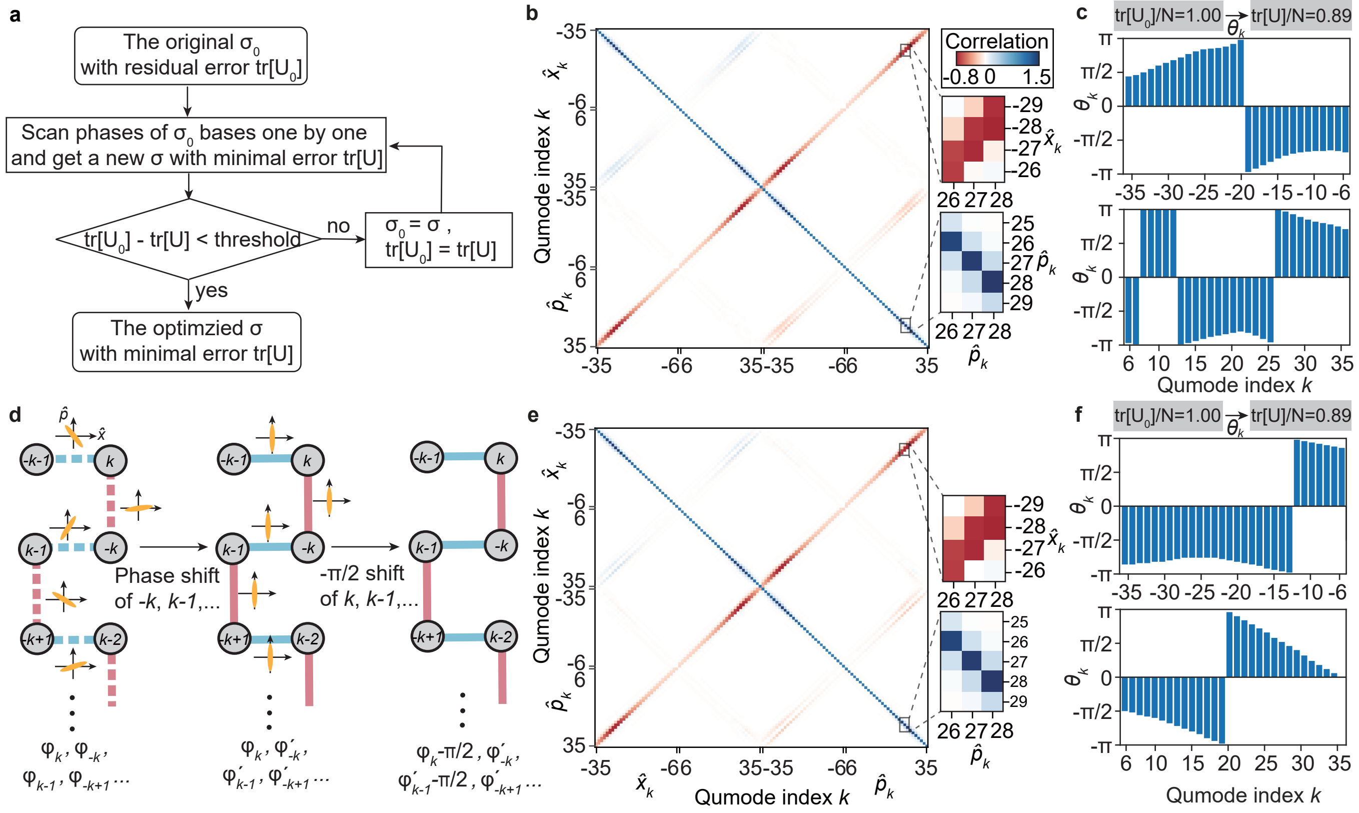

Fig. S2. **Phase realignment of 1D cluster state. a,** Working flow of global optimization. **b,** Covariance matrix after global optimization of the covariance matrix in Fig. S1a. **c,** Additional phase applied on each qumode after global optimization. **d,** Working flow of line-by-line optimization. **e,** Covariance matrix after line-by-line optimization of the covariance matrix in Fig. S1a. **f,** Additional phase applied on each qumode after line-by-line optimization.

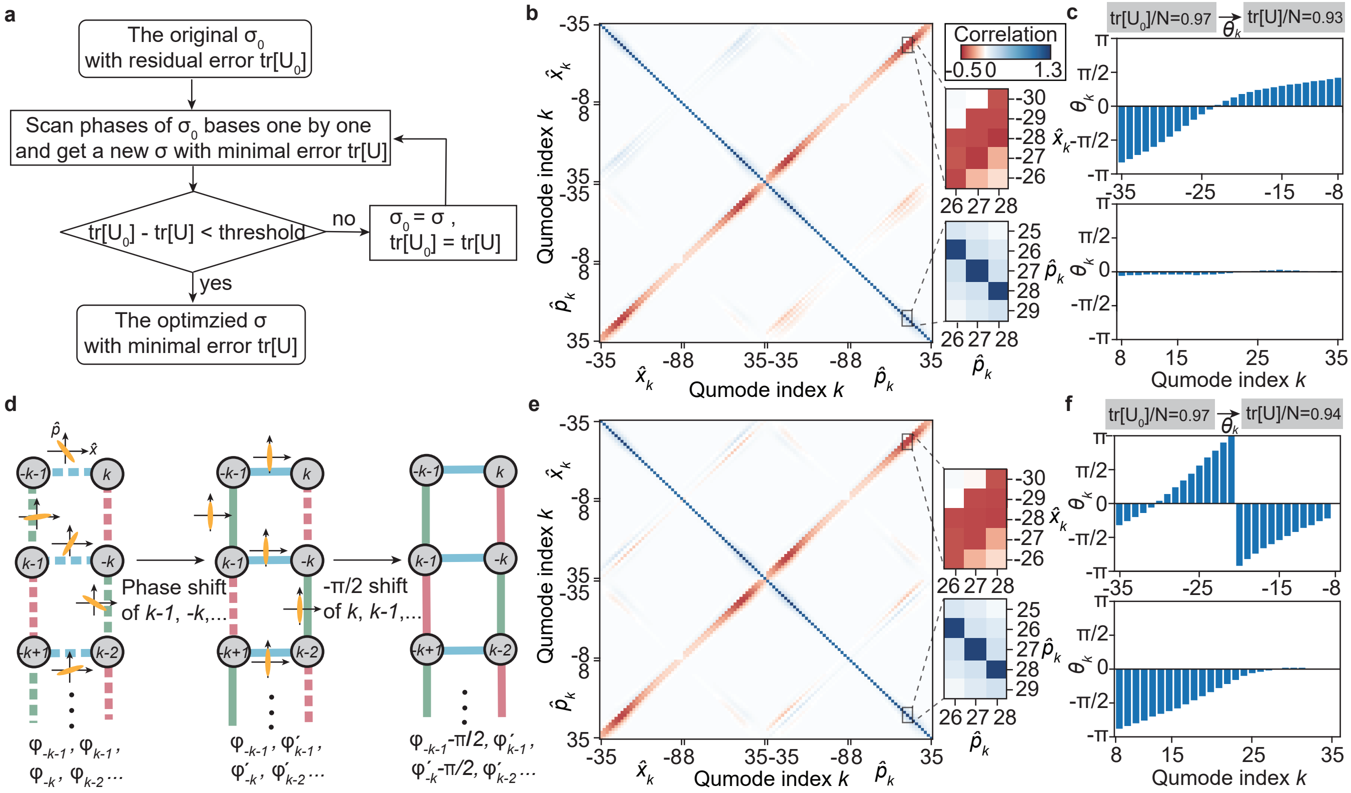

Fig. S3. **Phase realignment of 2D cluster state. a,** Working flow of global optimization. **b,** Covariance matrix after global optimization of the covariance matrix in Fig. S1c. **c,** Additional phase applied on each qumode after global optimization. **d,** Working flow of line-by-line optimization. **e,** Covariance matrix after line-by-line optimization of the covariance matrix in Fig. S1b. **f,** Additional phase applied on each qumode after line-by-line optimization.

4. *Impact of parasitic nonlinear optical processes*

In this section, we examine the influence of nonlinear optical processes other than the major TMS on the generated cluster state. Specifically, we consider minor TMS operations generated exclusively from secondary pump lines and the BS.

Firstly, we analyze the correlations induced by TMS operations. For TMS applied to qumodes n and m, the Hamiltonian is expressed as

$$\hat{\mathrm{H}}_{\mathrm{TMS}} = \hbar \left(G e^{i\theta} \hat{a}_n^\dagger \hat{a}_m^\dagger + \text{h.c.} \right) \tag{S40}$$

where the coupling rate $G > 0$. The covariance $\mathrm{Cov}(\hat{q}_i, \hat{q}_j)$ is computed using

$$\mathrm{Cov}(\hat{q}_i, \hat{q}_j) = \mathrm{Re}\left[(\langle \hat{a}_i \hat{a}_j \rangle + \langle \hat{a}_j \hat{a}_i \rangle) e^{i(\theta_i + \theta_j)} \right] + \mathrm{Re}\left[(\langle \hat{a}_i \hat{a}_j^\dagger \rangle + \langle \hat{a}_j^\dagger \hat{a}_i \rangle) e^{i(\theta_i - \theta_j)} \right] \tag{S41}$$

where $\hat{q}_i = \hat{a}_i e^{i\theta_i} + \hat{a}_i^\dagger e^{-i\theta_i}$ represents either the $\hat{x}_i$ or $\hat{p}_i$ quadrature with $\theta_i = 0$ or $\theta_i = \pi/2$, respectively. Based on the Hamiltonian, the correlations are calculated as

$$\mathrm{Cov}(\hat{q}_n, \hat{q}_m) = \sinh(2G) \cdot \mathrm{Re}\left[e^{i(-\theta - \pi/2 + \theta_n + \theta_m)} \right] \tag{S42}$$

When $\theta = \pi$, negative correlations emerge for $\mathrm{Cov}(\hat{p}_n, \hat{x}_m)$ and $\mathrm{Cov}(\hat{x}_n, \hat{p}_m)$, while $\mathrm{Cov}(\hat{x}_n, \hat{x}_m) = \mathrm{Cov}(\hat{p}_n, \hat{p}_m) = 0$.

The coupling rate G for minor TMS is typically less than 1/4 of that for major TMS under the pumping strategies described in the main text. Consequently, the correlations induced by minor TMS are reduced by a factor of less than 1/4 relative to those from major TMS. These effects are therefore negligible for the cluster states.

Next, we incorporate the BS into the analysis. The combined Hamiltonian is given by

$$\hat{\mathrm{H}}_{\mathrm{TMS+BS}} = \hbar \left(G_1 e^{i\theta_1} \hat{a}_n^\dagger \hat{a}_m^\dagger + G_2 e^{i\theta_2} \hat{a}_m^\dagger \hat{a}_l + \text{h.c.} \right) \tag{S43}$$

This leads to correlations between qumode pairs (n, l) as

$$\mathrm{Cov}(\hat{q}_n, \hat{q}_l) = f(G_1, G_2) \cdot \mathrm{Re}\left[e^{i(\theta_1 - \theta_2 + \pi + \theta_n + \theta_l)} \right] \tag{S44}$$

where

$$f(G_1, G_2) = \begin{cases} G_1^2 \left(1 + \dfrac{G_1^2}{2} \right), & \text{if } G_1 = G_2, \\ \dfrac{G_1 G_2}{\Delta G^2} \left[\dfrac{G_1^2 + G_2^2}{\Delta G^2} (\cosh \Delta G - 1)^2 + \sinh^2 \Delta G \right], & \text{if } G_1 \neq G_2, \end{cases} \tag{S45}$$

with $\Delta G = \sqrt{G_1^2 - G_2^2}$. When $\theta_1 - \theta_2 = \pi$, positive correlations are observed for $\mathrm{Cov}(\hat{x}_n, \hat{x}_l)$ and negative correlations for $\mathrm{Cov}(\hat{p}_n, \hat{p}_l)$, while the other covariances vanish.

The strength of these correlations depends on the BS coupling rate G_2. Using the pumping strategies described in the main text, G_2 approximately equals the major TMS coupling rate G_1. The level of G_1 is around 0.36 according to the measured correlation ($\sinh 2G_1 \approx 0.8$); in this regard, $f(G_1, G_2) \approx 0.14$, which is significantly smaller.

The theoretical analysis is verified for two scenarios: 1D and 2D cluster states. For both cases, the phases are realigned for the Hamiltonian including only the major TMSs to highlight the impact of the parasitic effects (thereby not exactly the phase settings used in experiments). In the case of a 1D cluster state, as illustrated in Fig. S4b, the introduction of minor TMS between qumode pairs $(k, -k-2)$ induces weak negative correlations in $\mathrm{Cov}(\hat{p}_k, \hat{x}_{-k-2})$. Additionally, as shown in Fig. S4c, the introduction of a BS between qumode pairs $(k, k \pm 1)$ results in weak positive correlations in $\mathrm{Cov}(\hat{x}_k, \hat{x}_{-k+1,+0,-1,-2})$, as well as weak negative correlations in $\mathrm{Cov}(\hat{p}_k, \hat{p}_{-k+1,+0,-1,-2})$.

For a 2D cluster state, as depicted in Fig. S5b, the introduction of minor TMS between qumode pairs $(k, -k+1)$ and $(k, -k+2)$ induces weak negative correlations in $\mathrm{Cov}(\hat{p}_k, \hat{x}_{-k+1})$ and $\mathrm{Cov}(\hat{p}_k, \hat{x}_{-k+2})$. Furthermore, as illustrated in Fig. S5c, the introduction of BS between qumode pairs $(k, k \pm 1)$ and $(k, k \pm 2)$ generates weak negative correlations in $\mathrm{Cov}(\hat{x}, \hat{x})$ and weak positive correlations in $\mathrm{Cov}(\hat{p}, \hat{p})$. These results are in good agreement with our theoretical predictions. Based on these analytical and numerical results, we conclude that for the pumping configurations presented in this work, the quantum correlations and graph structure are primarily determined by the major TMSs.

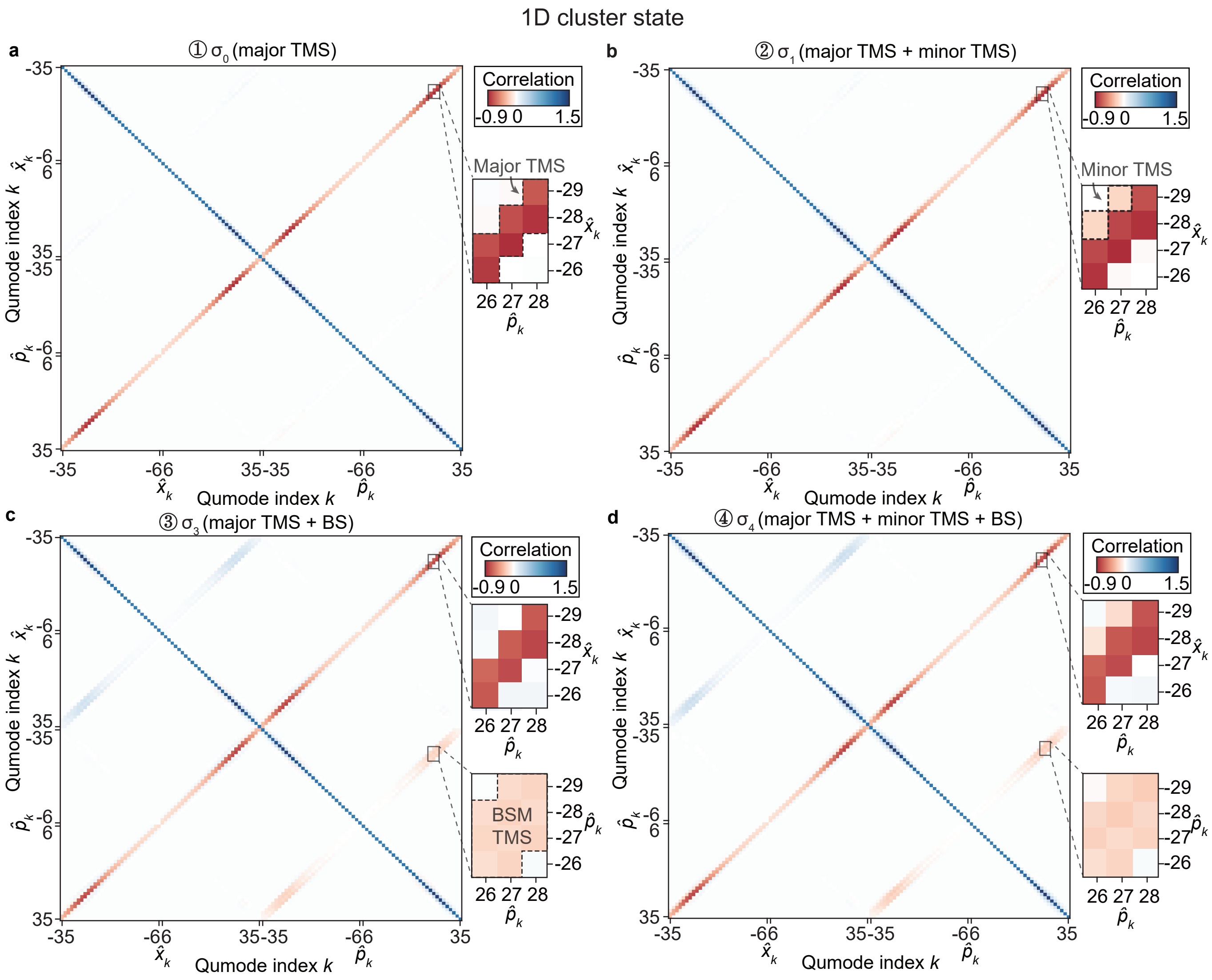

Fig. S4. **Impact of parasitic nonlinear processes on the 1D cluster state. a,** Covariance matrix considering only the major two-mode squeezing (TMS) operations. **b,** Covariance matrix including both major and minor TMS operations. **c,** Covariance matrix incorporating major TMS and Bragg scattering (BS) interactions. **d,** Covariance matrix accounting for all nonlinear terms.

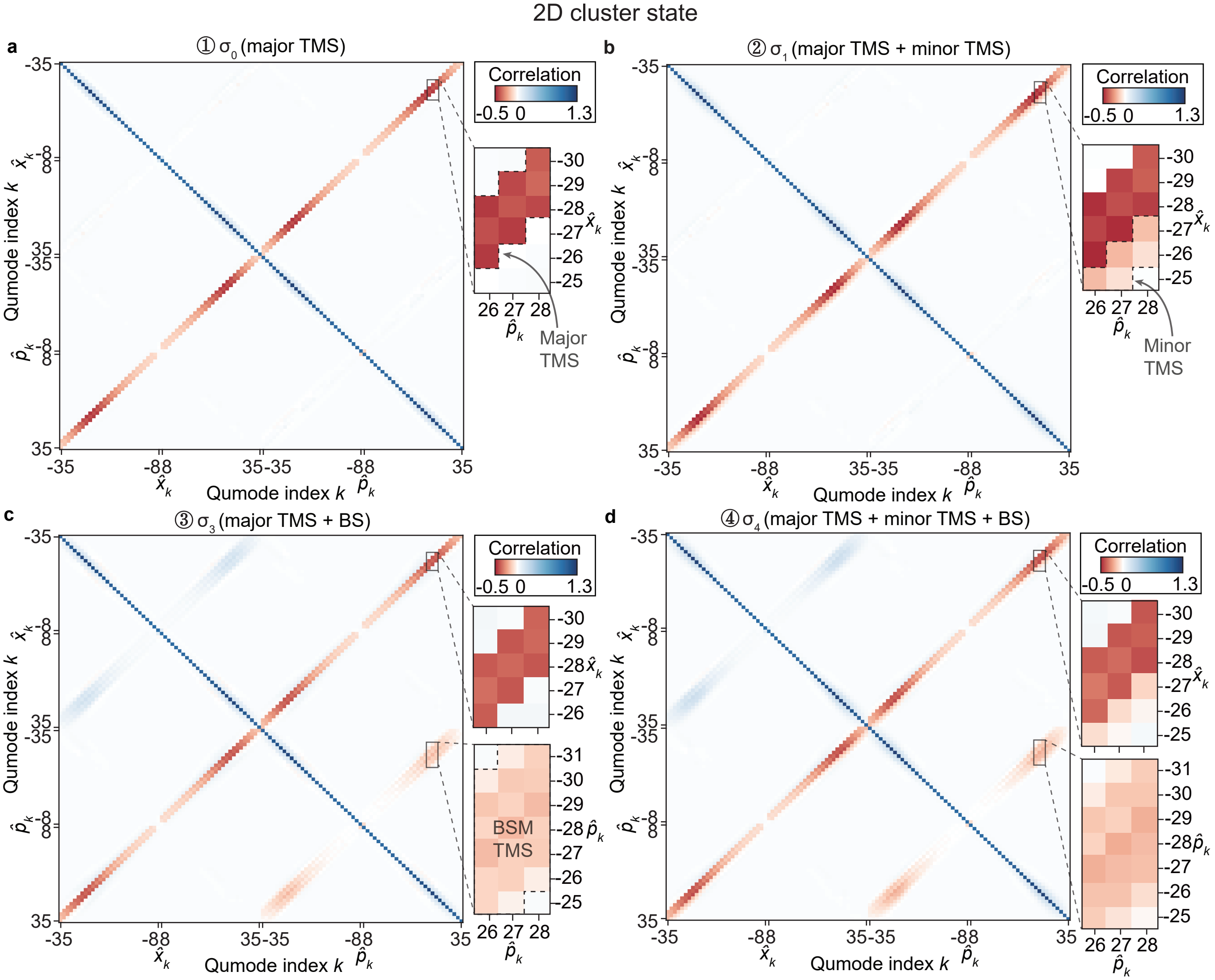

Fig. S5. **Impact of parasitic nonlinear processes on the 2D cluster state. a,** Covariance matrix considering only the major two-mode squeezing (TMS) operations. **b,** Covariance matrix including both major and minor TMS operations. **c,** Covariance matrix incorporating major TMS and Bragg scattering (BS) interactions. **d,** Covariance matrix accounting for all nonlinear terms.

Once the qumode phases are properly realigned, the graph structure is derived from the approximate nullifiers. We consider two cases: the 1D and 2D cluster states.

- **1D Cluster State.**

  The calculated adjacency matrix is shown in Fig. S6a. It displays a complex structure with additional edges beyond those introduced by major TMS operations, arising from minor TMS and BS interactions. Furthermore, the edge weights for major TMS are unequal, which is attributed to second-order dispersion effects.

  To address this complexity, we perform an additional optimization using alternative approximate nullifiers defined as

$$\hat{N}_k = \hat{p}_k + n\left(\hat{x}_{-k} + \hat{x}_{-k-1}\right) \tag{S46}$$

  The normalized residual error ($\mathrm{tr}\left[\mathrm{Cov}[\overline{N}] \cdot diag\{\dots,(1+\sum_n \mathbf{V}_{kn}^2),\dots\}^{-1}\right], \overline{N} = \overline{p} - \mathbf{V}\overline{x}$) for these nullifiers becomes smaller than that from the adjacency matrix when $n > 0.35$ (Fig. S6b), which is corresponding to nullifier squeezing. The minimal normalized residual error occurs at $n = 0.65$, achieving substantial squeezing compared to TMS (Fig. S6c).

  We compare the graph structures derived from the original adjacency matrix (Fig. S6d) and the optimized nullifiers (Fig. S6e). The structures are highly similar despite a few parasitic edges and variations in edge weights.

  Based on the residual error analysis, we select nullifiers with fixed weights of $n = 0.65$, resulting in a graph structure that conforms to a 1D lattice.

- **2D Cluster State.**

  The calculated adjacency matrix is presented in Fig. S7a. Similar to the 1D case, it exhibits additional edges from minor TMS and BS interactions, and unequal edge weights due to second-order dispersion effects.

  We perform an additional optimization using approximate nullifiers defined as

$$\hat{N}_k = \hat{p}_k + n\left(\hat{x}_{-k} + \hat{x}_{-k-1} + \hat{x}_{-k-2}\right) \tag{S47}$$

  which reduces the normalized residual error compared to the adjacency matrix for $0.25 < n < 0.95$ (Fig. S7b). The minimal normalized residual error occurs at $n = 0.5$, yielding substantial squeezing relative to TMS (Fig. S7c).

  We compare the graph structures derived from the original adjacency matrix (Fig. S7d) and the optimized nullifiers (Fig. S7e). The structures are highly similar despite a few parasitic edges and variations in edge weights.

  Based on the residual error analysis, we select nullifiers with fixed weights of $n = 0.5$, resulting in a graph structure that conforms to a 2D lattice.

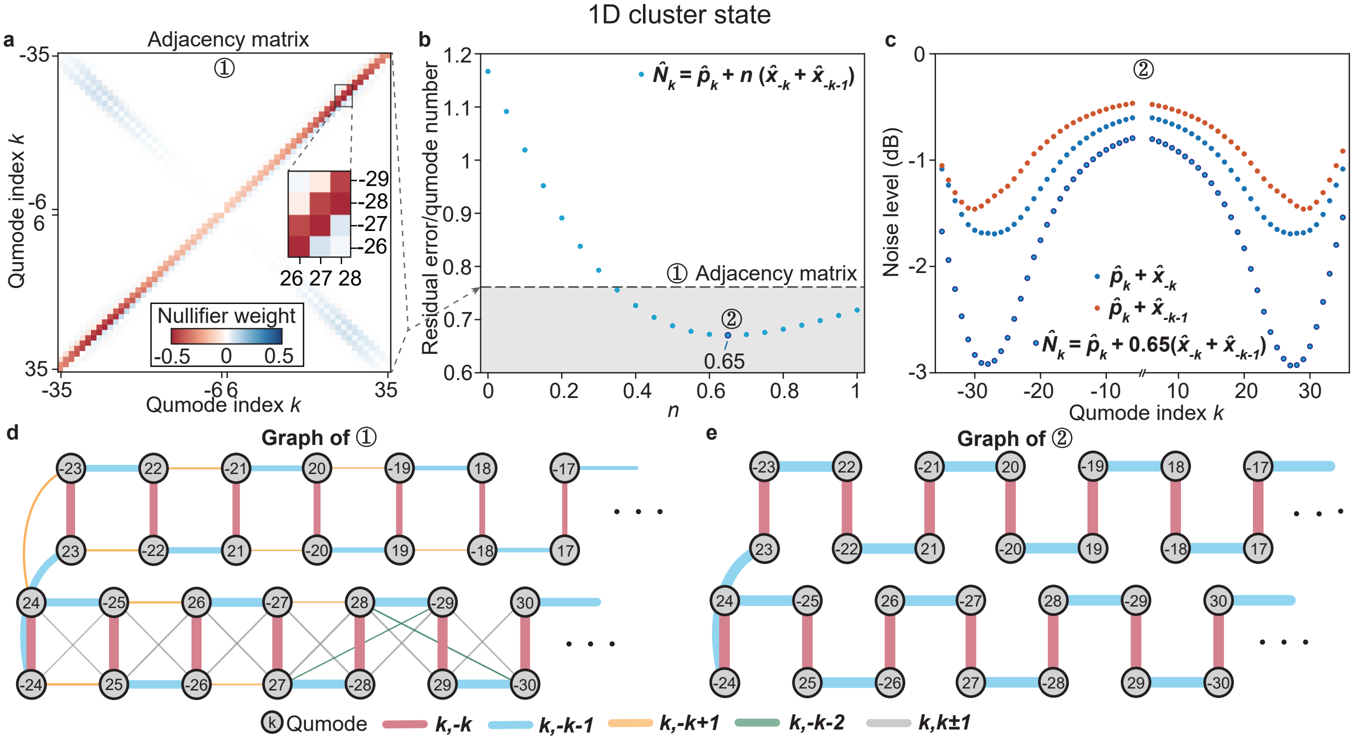

Fig. S6. **Approximate nullifiers and graph structures for the 1D cluster state. a,** Adjacency matrix corresponding to the covariance matrix depicted in Fig. S2b. **b,** Normalized residual error versus weight, with the dashed line indicating the error for the adjacency matrix in (a). **c,** Quadrature noise variances for selected operators. **d,** Graph structure derived from the adjacency matrix in (a), where edge widths represent the cluster state's weights; edges with weights within ± 0.05 are omitted. **e,** Graph structure of the optimized cluster state with a fixed edge weight of -0.65.

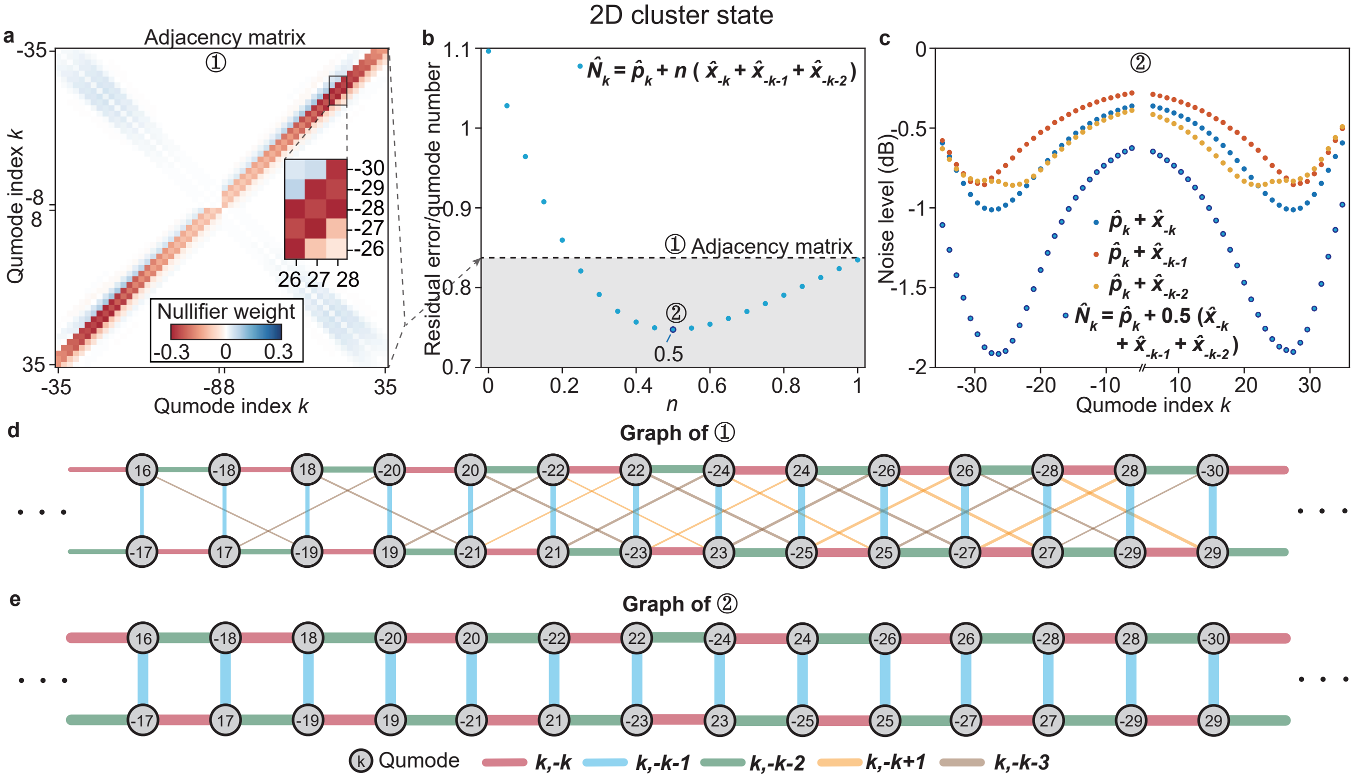

Fig. S7. **Approximate nullifiers and graph structures for the 2D cluster state.** **a,** Adjacency matrix corresponding to the covariance matrix depicted in Fig. S3b. **b,** Normalized residual versus weight, with the dashed line indicating the error for the adjacency matrix in (a). **c,** Quadrature noise variances for selected operators. **d,** Graph structure derived from the adjacency matrix in (a), where edge widths represent the cluster state's weights; edges with weights within ± 0.05 are omitted. **e,** Graph structure of the optimized cluster state with a fixed edge weight of -0.5.

E. Optimal pumping strategy

The pump lines are programmed to realize cluster states with optimal performance by maximizing correlations, equalizing edge weights, and minimizing parasitic nonlinear effects. Additionally, high power input into the microresonator can generate sidebands above the threshold, reducing the number of usable qumodes which should be avoided.

We first investigate the optimal pumping conditions for realizing a 1D cluster state by applying pump lines to modes 0 and -1. Intuitively, to achieve two sets of equal major TMSs, the secondary pump line should be 0.25 of the primary pump line. However, setting the secondary pump to be 0.2 also produces a similar covariance matrix regarding the negative correlations induced by major TMS (Fig. S8a). In addition, it generates fewer lasing sidebands for the same level of the primary pump line (Fig. S8b), thereby increasing the number of usable qumodes. Consequently, we choose a $1 : 0.2$ ratio in the experiment.

Next, we consider realizing a 2D cluster state by applying pump lines to modes -1, 0, and 1. The coupling rates of the three major TMS operations are given by $A_{-1}A_{-1}$, $2A_{-1}A_0$, and $2A_{-1}A_1 + A_0A_0$. Based on the analysis above, we set $|A_0|^2$ to 0.2 of $|A_{-1}|^2$, and subsequently set $|A_1|^2$ to 0.16 of $|A_{-1}|^2$ to equalize these major TMSs. We adopt this configuration to generate 2D cluster states in the experiment.

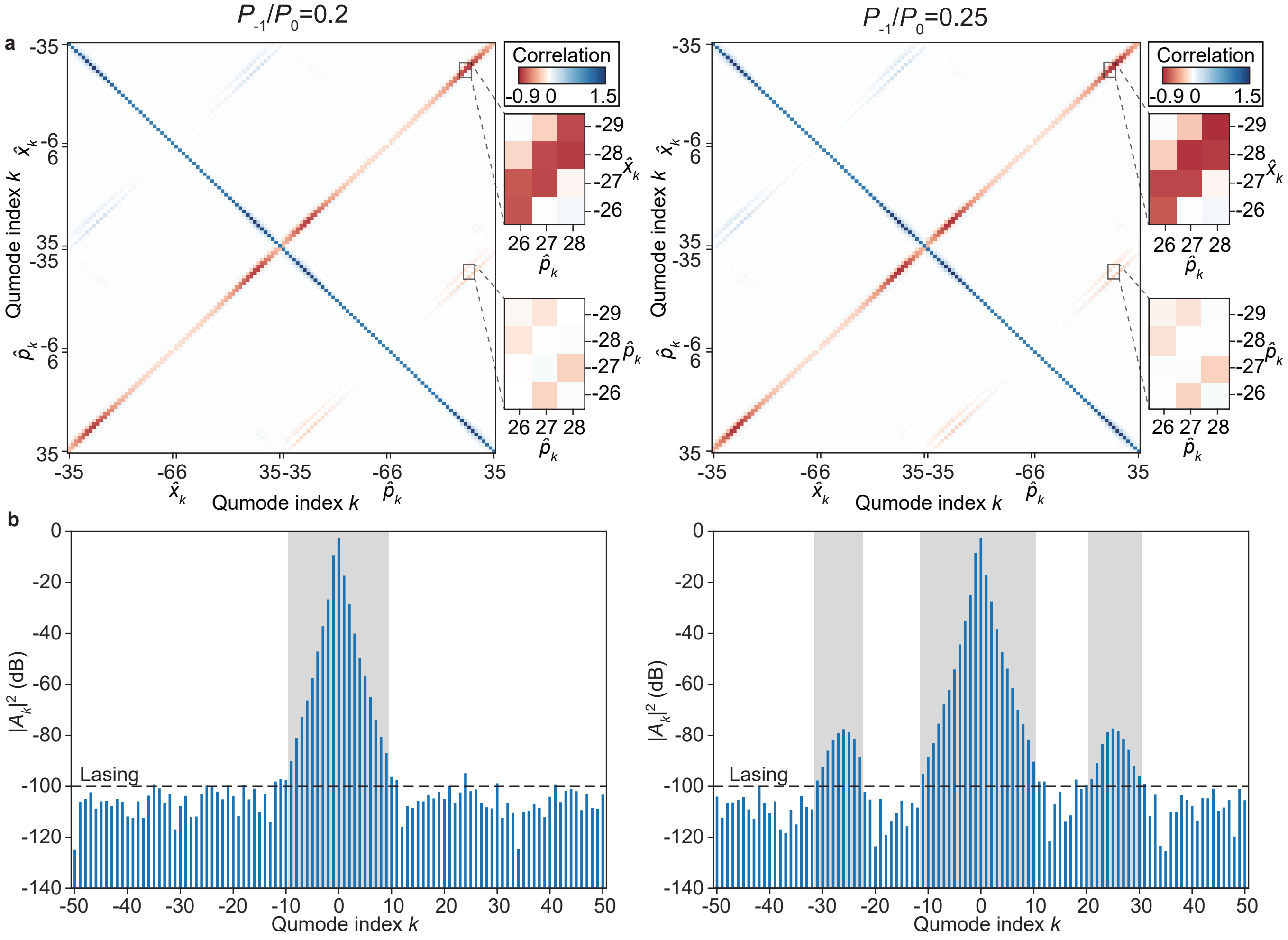

Fig. S8. **Comparison of different pumping strategies. a,** Covariance matrices of pump power ratio $P_{-1}/P_0 = 0.2$(left) and $P_{-1}/P_0 = 0.25$(right). **b,** Spectrum of pump power ratio $P_{-1}/P_0 = 0.2$(left) and $P_{-1}/P_0 = 0.25$(right). Gray shades indicate qumodes whose amplitude $|A_k|^2$ are over threshold. Parameters used in the simulations are: $P_0 = 0.7P_{\rm th}$, $D_2 = 0.0026\kappa$, $\zeta_0 = -0.75\kappa$ for (a) and $\zeta_0 = 0.21\kappa$ for (b), and $\eta_e = \eta_d = 1$.

II. EXPERIMENTAL DETAILS

A. Device fabrication

The microresonators are fabricated on a high-resistivity silicon wafer coated with an 8-μm-thick thermally oxidized silica film. Initially, the wafer is spin-coated with a photoresist (S1813) to a nominal thickness of 1.4 μm, followed by ultraviolet lithography using a contact mask aligner (SUSS MA6). The photoresist patterns are transferred to the underlying silica film via a wet etching process employing buffered oxide etchant for 150 minutes. After removing the photoresist, the silicon substrate is etched using XeF_2 gas to suspend the disk structure. Finally, the microresonators undergo annealing in a flowing argon environment at 950 °C for 30 minutes using a rapid thermal annealer. This annealing step reduces absorption losses and enhances the quality factor of the microresonators.

B. Characterization of dispersion and linewidth

A tunable continuous-wave laser (New Focus, TLB-6700) is utilized to characterize the dispersion and linewidth of the longitudinal mode family. The laser's relative frequency is calibrated using a Mach-Zehnder interferometer. Fitting the dispersion curve yields a second-order dispersion parameter of $D_2/2\pi = 23.9$ kHz (Fig. S9a). In addition to the targeted mode family, other transverse mode families are observed, locally altering the dispersion characteristics. The linewidths of the targeted modes, ranging from 1540 nm to 1560 nm, are extracted and found to be greater than ten times the intrinsic linewidth (Fig. S9b). This ensures an extraction efficiency exceeding 90% across the entire spectral range utilized in this study.

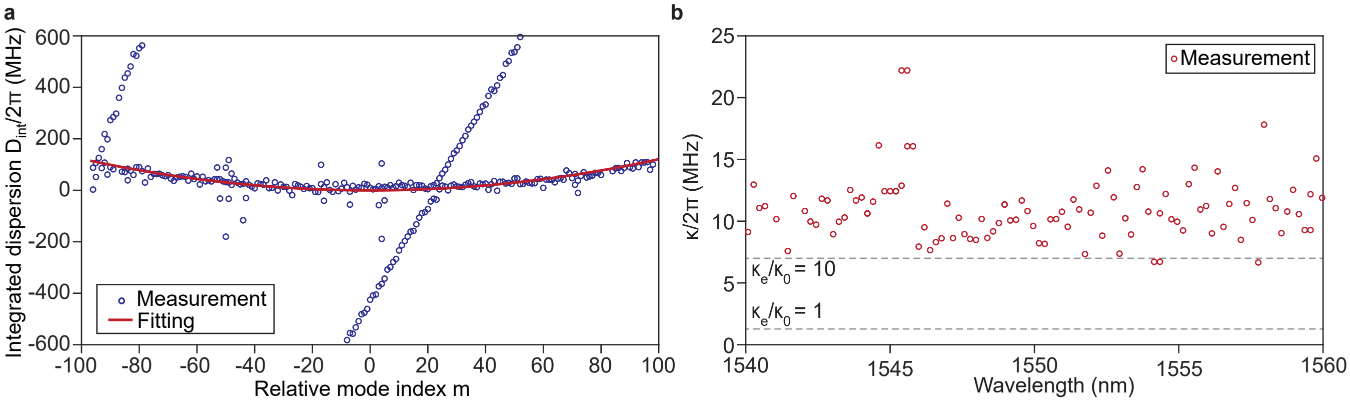

Fig. S9. **Characterization of the longitudinal mode family. a,** Mode familty dispersion. The integrated dispersion is defined as $D_{\mathrm{int}}(\mu) = \omega_\mu - \mu D_1 = D_2\mu^2/2 + \mathcal{O}(\mu^3)$ as a function of mode index μ relative to the pump. ω_μ is the resonant frequency of the μ_{th} mode and D_1 is the FSR. The red line denotes parabolic fitting with $D_2/2\pi$=23.9 kHz. **b,** Distribution of the total loss rates (κ) of the longitudinal mode family versus wavelength. The total loss rate corresponding to critical coupling ($\kappa_e/\kappa_0 = 1$) and strong over-coupling ($\kappa_e/\kappa_0 = 10$) is also indicated.

C. Experimental setup

The detailed experimental setup is illustrated in Fig. S10. The continuous-wave laser operates at approximately 1550.06 nm (New Focus, TLB-6700). A microwave generator (Keysight PSG E8257D) produces low-noise 25 GHz microwave signals, which are amplified to drive the modulators. For phase modulation, the phases of the microwave signals are adjusted to achieve the broadest spectrum. In contrast, for intensity modulation, the microwave phases and direct current (DC) bias are optimized to flatten the comb spectrum. The electro-optic (EO) comb power is tapped using a 90/10 fiber directional coupler, with 10% of the power monitored via a photodetector for servo-locking the DC bias of the intensity modulator, thereby ensuring long-term stability. A multi-channel programmable filter (II-IV Waveshaper 4000A) with a spectral resolution of 10 GHz synthesizes the pump and local oscillator (LO) from the EO comb.

The pump is amplified using an erbium-doped fiber amplifier (EDFA), with amplified spontaneous emission (ASE) noise collectively filtered by a fixed-wavelength wavelength division multiplexer and a tunable bandpass filter. The transmitted optical signal from the microresonator is tapped using a 99/1 fiber directional coupler, with 1% of the light monitored by a photodetector. This signal is utilized to feedback-control the pump laser frequency, maintaining sustained long-term operations.

The LO is amplified using an EDFA and then combined with the transmitted signal from the microresonator via a 50/50 fiber directional coupler, achieving a high interference efficiency of 99.7%. To minimize dispersion effects, the temporal waveforms of the EO comb at the coupler input are interrogated using an autocorrelator. Proper group velocity dispersion, applied via the programmable filter, ensures the minimal temporal width of the EO comb, indicating a flat phase across all comb lines.

We implemented a homemade tunable filter within a 4-f optical system, comprising two diffraction gratings and two lenses, to eliminate the residual pump. The diffraction gratings have a linear density of 1200 lines$\cdot\mathrm{mm}^{-1}$ and an absolute diffraction efficiency exceeding 94.5%. The combined optical signals are collimated and spatially dispersed by the first grating. This filter effectively removes residual pump lines with a bandwidth of 200 GHz and a spatial resolution of 125 GHz$\cdot\mathrm{mm}^{-1}$, while other wavelengths are transmitted with minimal loss and subsequently recombined by the second grating. The overall transmission efficiency of our tunable filter is approximately 90%. After the filter, the remaining light is detected by a high-quantum-efficiency (92.5%) homemade balanced photodetector (Fermionics, FD500N-1550), whose noise performance is characterized in Fig. S10c. The balanced photodetector operates in the shot-noise-limited regime with an input power exceeding 2 mW and exhibits a common-mode noise rejection of 35 dB. All electrical spectra are recorded using an electrical spectrum analyzer (ESA, Rohde & Schwarz FPL1026). The overall detection efficiency of our system, accounting for 20% optical loss, 92.5% photodiode quantum efficiency, and 97% transmission of the tapered fiber, is 71.8%.

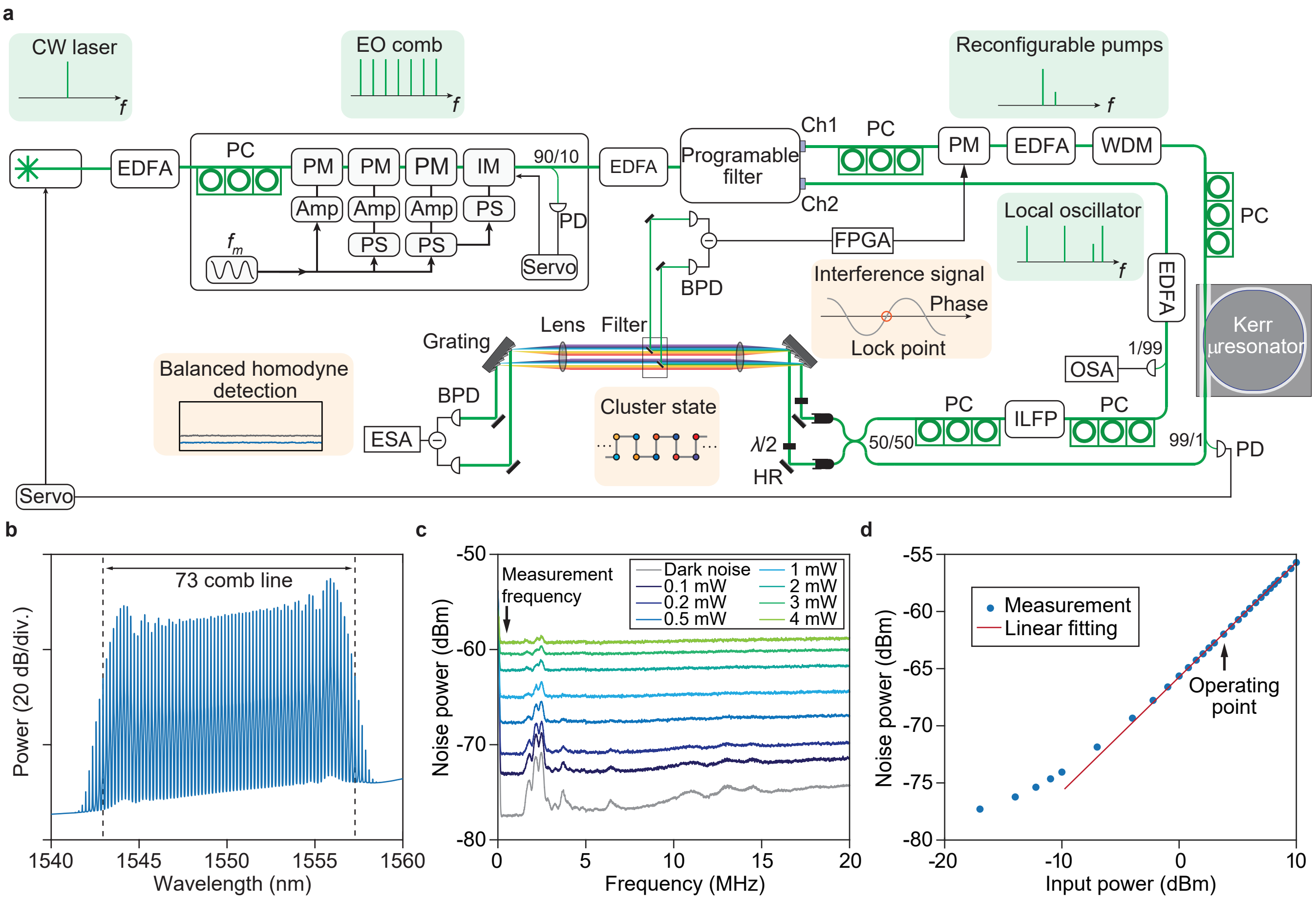

Fig. S10. **Detailed experimental setup and device performance. a,** Experimental setup. CW laser: Continuous-wave laser; EO comb: Electro-optic comb; EDFA: Erbium-doped fiber amplifier; PC: Polarization controller; PM: Phase modulator; IM: Intensity modulator; AMP: Electrical amplifier; PS: Electrical phase shifter; WDM: wavelength division multiplexer; FPGA: Field programmable gate array; ILFP: In-line fiber polarizer; HR: mirror with highly-reflective coating; $\lambda/2$: half-wave plate; PD: Photodetector; BPD: Balanced photodetector; ESA: Electrical spectrum analyzer; OSA: Optical spectrum analyzer. **b,** The optical spectrum of the EO comb. **c,** Electrical noise spectra of the BPD at different input optical power. **d,** Electrical noise of the BPD at 0.5 MHz as a function of input optical power. The red line indicates the linear operating regime. All measurements are taken with a resolution bandwidth of 100 kHz and a video of 100 Hz.

D. Phase-locked balanced homodyne detection

A phase-locking setup to stabilize the relative phase between the local oscillator (LO) and the pump is depicted in Fig. S10a. The comb line corresponding to the primary pump is consistently routed into the LO channel via the programmable filter, enabling subsequent interference with the primary pump in the pump channel. The mixed residual pump signal from both channels is extracted using a custom tunable filter and detected by a balanced photodetector. This signal is employed to lock the DC bias of a phase modulator, which regulates the phase of the pump entering the microresonator. Applying a 6 V DC bias to the phase modulator induces a 2π phase shift. However, typical proportional-integral-derivative (PID) controllers are unsuitable for this application because phase drifts can far exceed 2π, surpassing the servo's output limits. Consequently, we utilize a field-programmable gate array (FPGA) as the servo to realize cyclic PID control for an extended locking range. For example, if the output exceeds 3 V, the FPGA applies an instantaneous shift of -6 V to the output signal to keep it within the range of -3 V to 3 V, ensuring continuous phase-locking operation.

The reliability of the cyclic phase-locking is verified by monitoring the fluctuations in quadrature noise variance of the qumode pair (-17, 17) under a monochromatic pump. As shown in Fig. S11, the variance remains stable upon activation of the phase lock. This phase-locking setup enables precise measurement of the noise variance of the quadratures $\hat{q}$ and their linear combinations, thereby facilitating accurate determination of the covariance matrix.

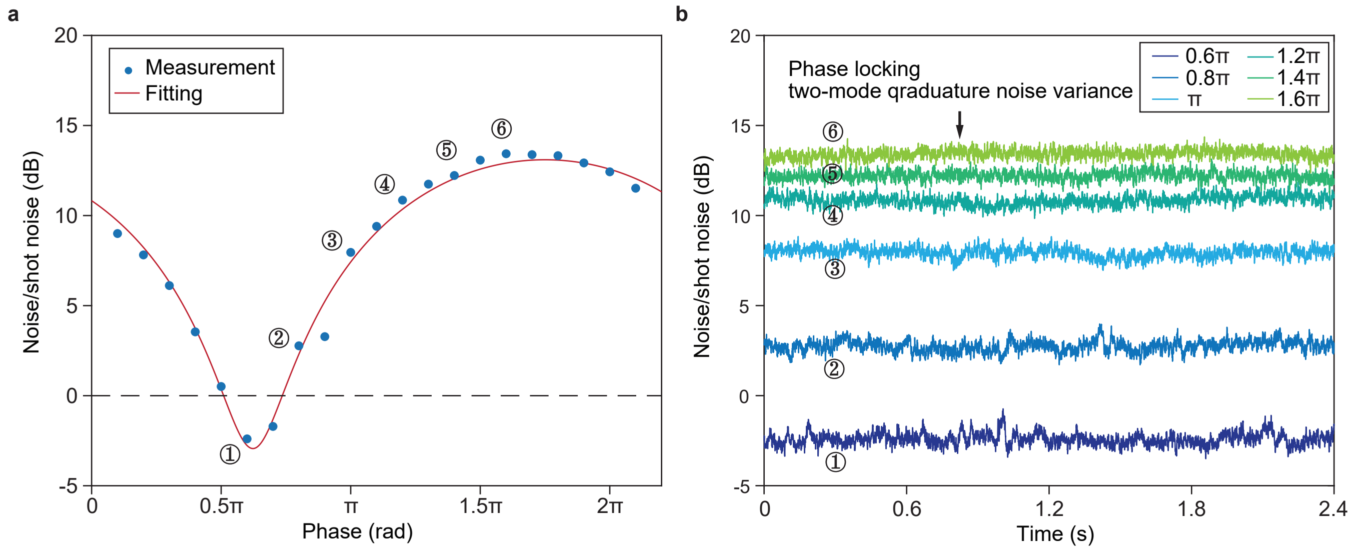

Fig. S11. **Phase locking performance. a,** Quadrature noise variance for qumode pair $(-16,\ 16)$ using a monochromatic pump (blue points) relative to shot noise (black dashed line) as a function of the relative phase between the local oscillator and the pump. The red line represents the fitting curve, indicating $3.09\,\mathrm{dB}$ of squeezing. **b,** Electrical spectra showing quadrature noise variance at various relative phases between the LO and pump when the phase locking remains activated.

E. Covariance matrix measurement

As outlined in Eq. (S11), measuring the covariance matrix involves two components: the noise variance of $\frac{1}{\sqrt{2}}\left(\hat{q}_m + \hat{q}_n\right)$ and the individual variances of $\hat{q}_m$ and $\hat{q}_n$. To accurately acquire the quadrature noise variance, it is essential to ensure that the LO components at the two qumodes are equal. This is achieved through an automatic spectral shaping system.

To implement spectral shaping, 1% of the LO light is tapped into a photodetector before entering the custom filter, and its spectral power is monitored using an optical spectrum analyzer (OSA). Automatic spectral shaping is performed using a programmable filter in conjunction with the LO light spectrum obtained from the OSA (Fig. S12a). The power difference ΔP between LO light lines at modes m and n is determined from the OSA data. An additional attenuation proportional to ΔP is applied to the LO light line with higher power via the programmable filter. This process is iterated up to three times or until ΔP becomes sufficiently small (Fig. S12b).

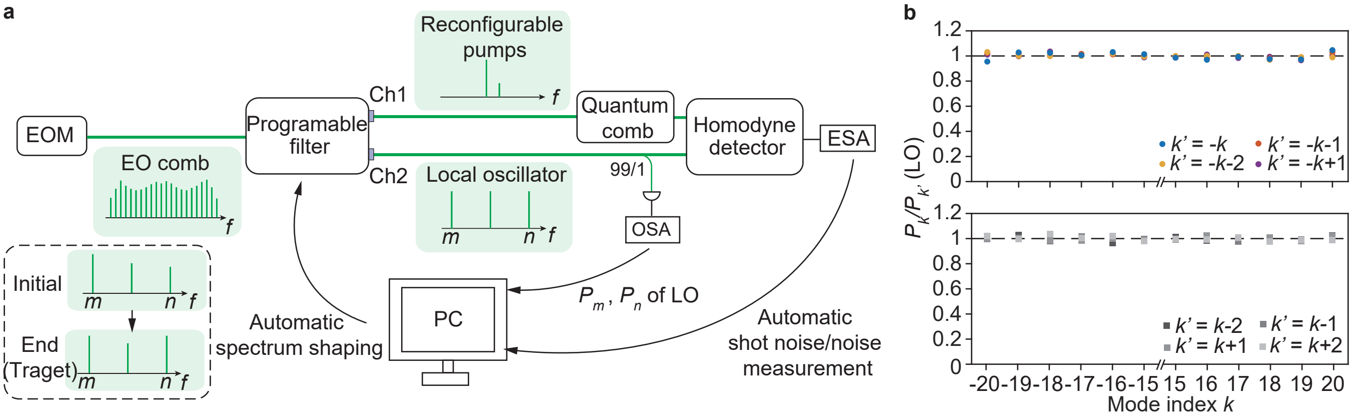

Fig. S12. **Measurement of the covariance matrix. a,** Protocol for automatic spectral shaping. **b,** Power ratio between two LO lines after spetral shaping.

III. ADDITIONAL MEASUREMENTS

A. Raw squeezing of EPR pairs

Within the 1.79-THz optical span of the EO comb, we measure the quadrature noise variance of 32 EPR pairs generated using the primary pump line only, with raw data summarized in Fig. S13. The spectral dependence is primarily attributed to the parametric gain. However, raw squeezing around qumodes (-23, 23) exhibited abnormal degradation, attributed to avoided mode crossing caused by coupling with other transverse mode families co-existing in the microresonator as shown in Fig. S9a.

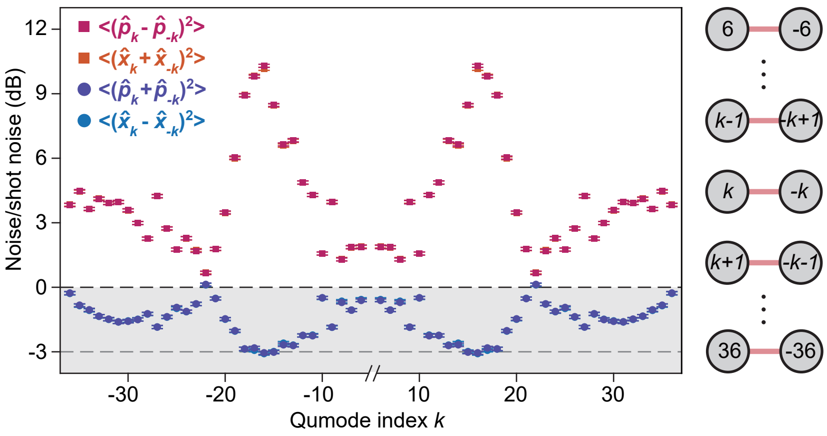

Fig. S13. **Measurement of EPR pairs generated by monochromatic pump laser.** Measured quadrature noise variances for EPR pairs $(k, -k)$. The right panel shows the entanglement structure.

B. Zero check of covariance matrix

Acquiring the covariance matrix is time-consuming, scaling as N^2, where N is the number of qumodes. To expedite this process, we selectively measure only the elements expected to exhibit non-zero correlations. Theoretical and numerical analyses (Fig. S1) indicate that, for a 1D cluster state, the covariance matrix elements are significantly non-zero for qumode pairs (k, k), $(k, k \pm 1)$, $(k, k \pm 2)$, $(k, -k)$, $(k, -k \pm 1)$, and $(k, -k - 2)$. For a 2D cluster state, the correlated qumode pairs include (k, k), $(k, k \pm 1)$, $(k, k \pm 2)$, $(k, -k)$, $(k, -k \pm 1)$, $(k, -k - 2)$, and $(k, -k - 3)$. To experimentally validate this selection, we perform zero-checks on all covariance matrix elements related to qumode -17, as summarized in Fig. S14 and Fig. S15. We find that only the elements selected for display in the main text exhibit significantly non-zero correlations, confirming the appropriateness

of our selection.

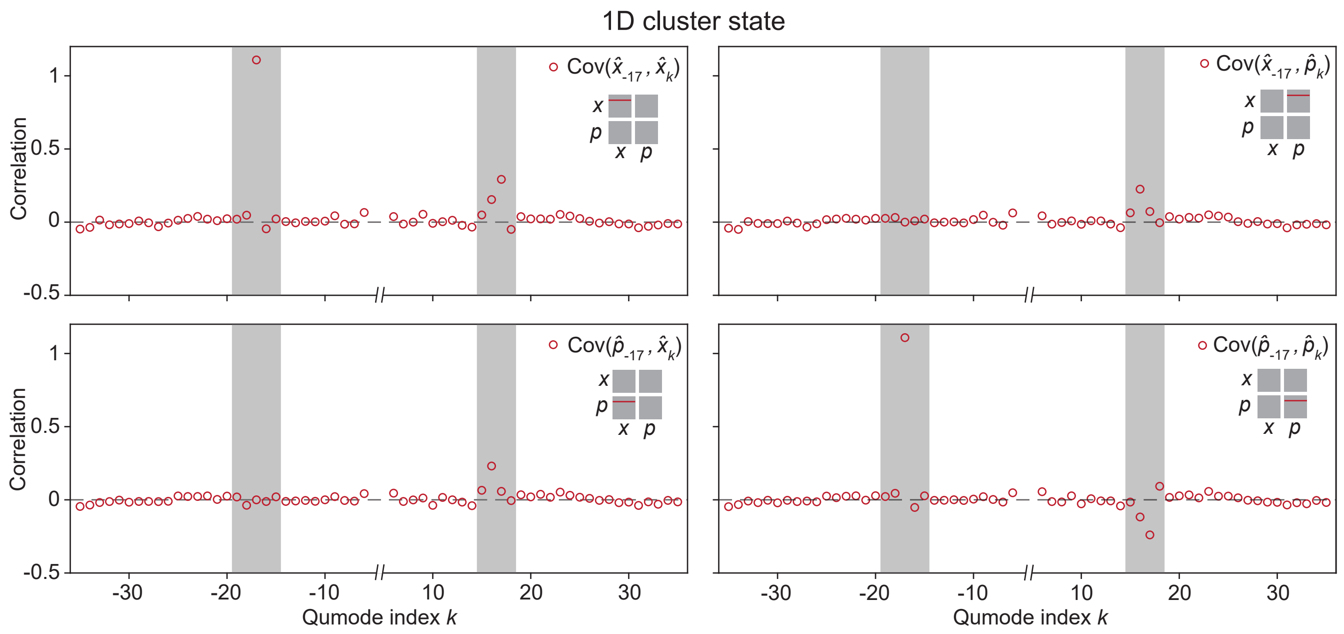

Fig. S14. **Zero-checks for 1D cluster state.** Quantum correlation related to qumode -17. The gray shades indicate elements displayed in the main text.

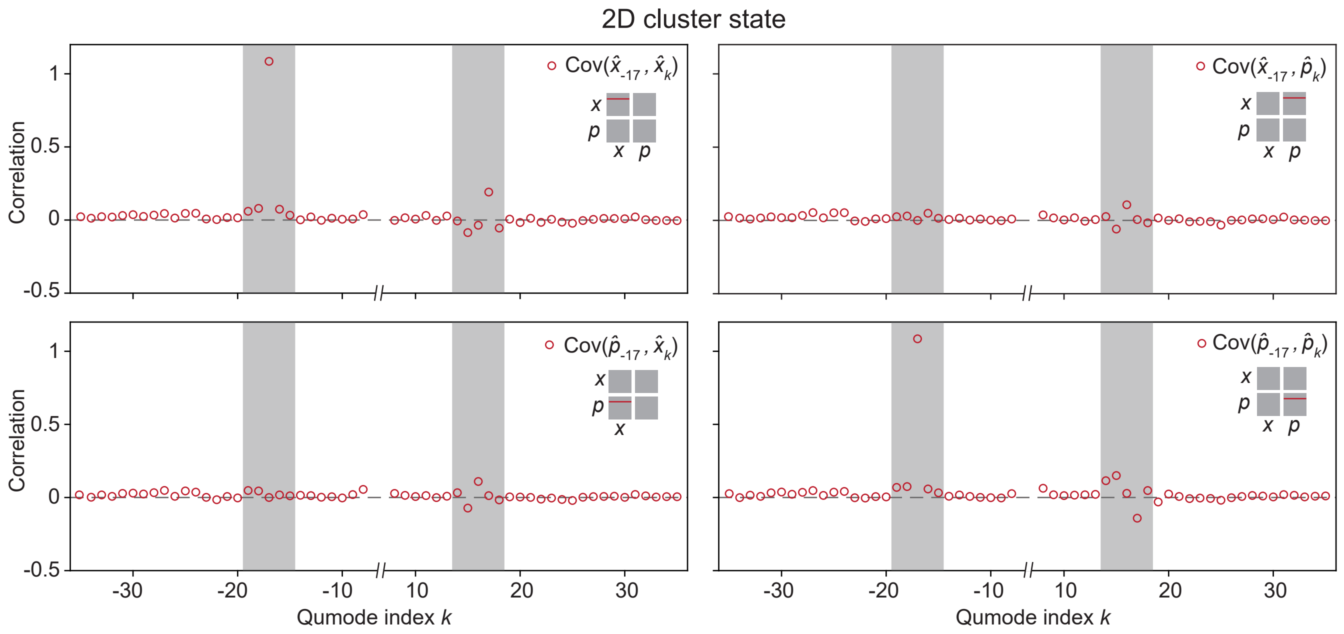

Fig. S15. **Zero-checks for 2D cluster state.** Quantum correlation related to qumode -17. The gray shades indicate elements displayed in the main text.

C. Realignment of qumode phases: experiment

The experimentally acquired covariance matrix for the 1D cluster state is shown in Fig. S2a. After applying phase realignment using a global optimization approach, the covariance matrix conforms to the standard form suitable for analyzing the cluster state

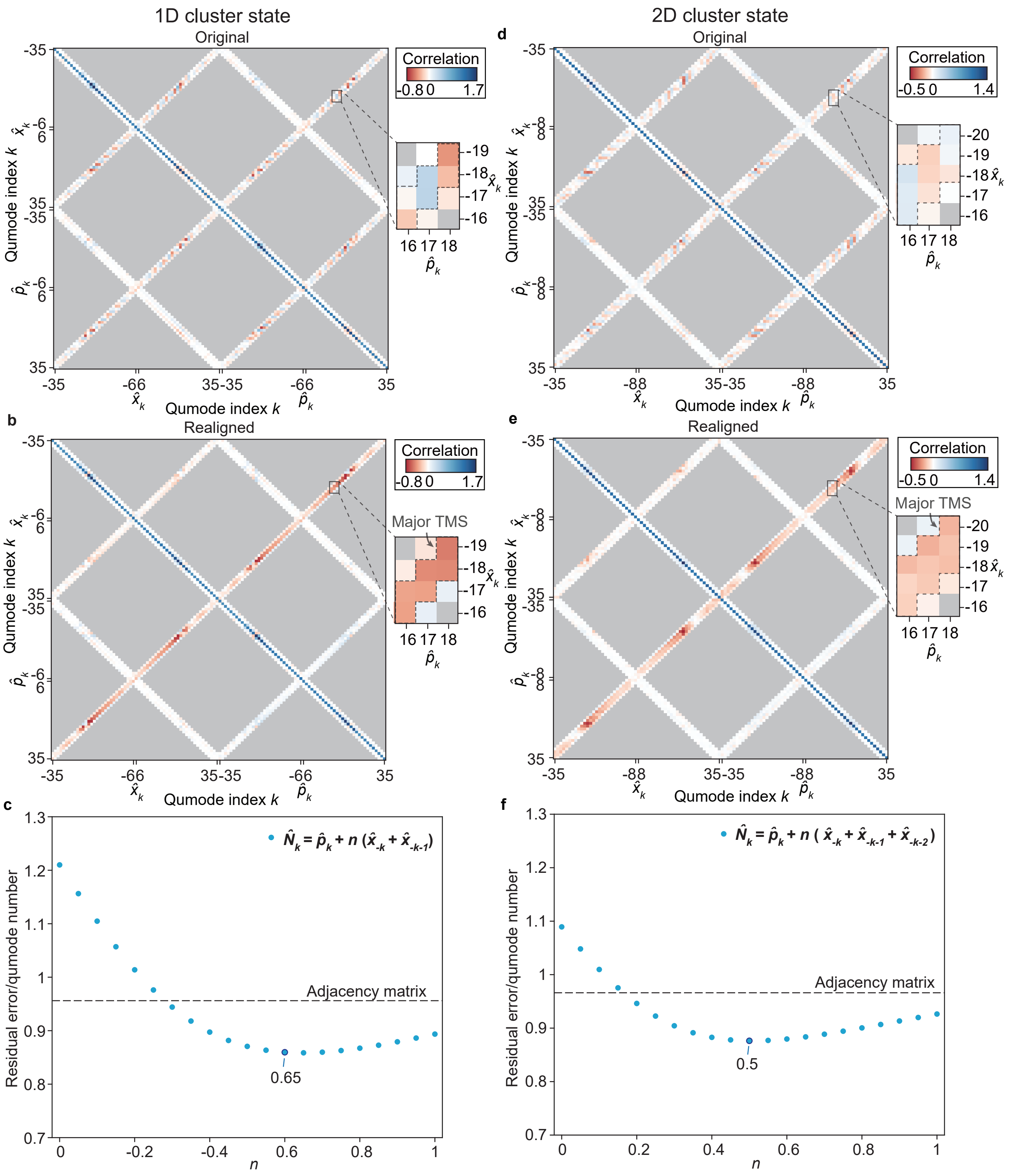

Fig. S16. **Realignment of qumode phases in 1D and 2D cluster state. a,** Measured covariance matrix for 1D cluster state. **b,** Covariance matrix by phase realignment of (a). **c,** Residual error as a function of edge weight for 1D cluster state. The dashed line indicates residual error derived from the adjacency matrix. **d,** Measured covariance matrix for 2D cluster state. **e,** Covariance matrix by phase realignment of (c). The measurements in the gray shade are not taken. **f,** Residual error as a function of edge weight for 2D cluster state. The dashed line indicates residual error derived from the adjacency matrix.

(Fig. S2b). Similarly, Figs. S2d and E display the original and realigned covariance matrices for the 2D cluster state, respectively.

To determine the graph structure, we calculate the residual errors for nullifiers with varying weights. We find that for the 1D cluster state, a good approximation of the nullifiers is $\hat{p}_k + n(\hat{x}_{-k} + \hat{x}_{-k-1})$, with $n = 0.65$ minimizing the residual error. Hence, in the experiment, we choose $\hat{p}_k + 0,65(\hat{x}_{-k} + \hat{x}_{-k-1})$. For the 2D cluster state, an approximate set of nullifiers is $\hat{p}_k + n(\hat{x}_{-k} + \hat{x}_{-k-1} + \hat{x}_{-k-2})$, with $n = 0.5$ yielding a minimum residual error. Therefore in the experiment we choose $\hat{p}_k + 0.5(\hat{x}_{-k} + \hat{x}_{-k-1} + \hat{x}_{-k-2})$.

IV. QUANTUM FISHER INFORMATION

In classical parameter estimation theory, the Cramér-Rao bound provides a fundamental lower limit on the variance of any unbiased estimator of a parameter θ, expressed as

$$\mathrm{Var}(\theta_{\mathrm{est}}) \geq \frac{1}{F_c(\theta)} \tag{S48}$$

where $F_c(\theta)$ denotes the Fisher information associated with the parameter θ.

In the quantum domain, parameters can be encoded into quantum states, and their values can be estimated through appropriate measurements. Quantum theory posits the existence of an optimal measurement strategy that maximizes the Fisher information, thereby defining the quantum Fisher information (QFI). The QFI represents the ultimate precision limit for parameter estimation in quantum mechanics [2]. It is given by

$$F_Q(\theta) = \mathrm{Tr}[\rho(\theta)\hat{L}^2(\theta)] \tag{S49}$$

where $\hat{L}(\theta)$ is the symmetric logarithmic derivative associated with the quantum state $\rho(\theta)$.

Focus on n-mode Gaussian states, which are fully characterized by their first and second moments, and consider a set of quadrature operators in phase space, $\hat{\mathbf{r}} = (\hat{x}_1, \hat{p}_1, \ldots, \hat{x}_n, \hat{p}_n)^\top$, which defines the covariance matrix σ. To encode a parameter θ, we apply a displacement operator $\hat{D}(\theta) = \exp(-i\theta\hat{q}(\mathbf{g}))$, where $\hat{q}(\mathbf{g}) = \mathbf{g} \cdot \hat{\mathbf{r}}$ and $\mathbf{g} = (g_1, \ldots, g_n)^\top$. The QFI for the displaced Gaussian state is then calculated as [3]

$$F_Q[\rho, \hat{q}(\mathbf{g})] = \mathbf{g}^\top \mathbf{\Omega}^\top \sigma^{-1} \mathbf{\Omega} \mathbf{g} \tag{S50}$$

where $\mathbf{\Omega} = \bigoplus_{i=1}^{n} \begin{pmatrix} 0 & 1 \\ -1 & 0 \end{pmatrix}$ is the symplectic form.

To maximize the metrological power, we can control the relative phases of the output quantum state by rotating the measurement basis. This rotation aligns the quantum correlations such that squeezing manifests in linear combinations of the $\hat{x}_k$ quadratures. Consequently, the parameter θ is encoded by displacing the cluster quantum microcombs along the $\hat{x}$ quadratures, corresponding to $\mathbf{g} = (0, 1, \ldots, 0, 1)^\top$.

We calculate the QFI for both 1D and 2D cluster state configurations, as depicted in Fig. S17. The results demonstrate that, in both scenarios, the QFI increases with the number of qumodes (start from qumodes $\pm 19, \pm 20$ and increase towards both sides), surpassing the standard quantum limit. Specifically, for an n-mode coherent state, the standard quantum limit is associated with $F_{\mathrm{SQL}} = n$. Our cluster quantum microcombs achieve QFI values exceeding F_{SQL}, indicating enhanced metrological capabilities.

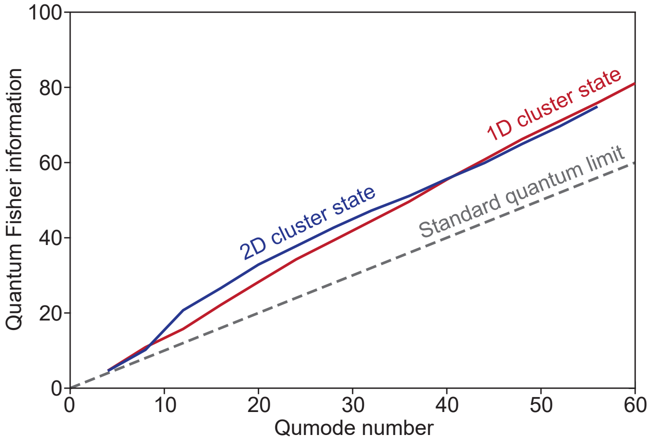

Fig. S17. **Quantum Fisher information in 1D cluster state and 2D cluster state.** Experimental quantum Fisher information (QFI) for the 1D (red solid line) and 2D (blue solid line) cluster states, compared to the standard quantum limit (gray dashed line).

[1] Chembo, Y. K. Quantum dynamics of Kerr optical frequency combs below and above threshold: Spontaneous four-wave mixing, entanglement, and squeezed states of light. *Phys. Rev. A* **93**, 033820 (2016).

[2] Pezzè, L., Smerzi, A., Oberthaler, M. K., Schmied, R. & Treutlein, P. Quantum metrology with nonclassical states of atomic ensembles. *Rev. Mod. Phys.* **90**, 035005 (2018).

[3] Qin, Z. *et al.* Characterizing the multipartite continuous-variable entanglement structure from squeezing coefficients and the fisher information. *npj Quantum Inf.* **5**, 3 (2019).
